# Who Reports Greater Chronic Disease Risk Behaviours? A Closer Look at Sociodemographic Differences Among Australian Adolescents: A Cross‐Sectional Analysis

**DOI:** 10.1002/hpja.70079

**Published:** 2025-07-15

**Authors:** Lyra Egan, Siobhan O’Dean, Lauren A. Gardner, Nicola C. Newton, Katrina E. Champion

**Affiliations:** ^1^ The Matilda Centre for Research in Mental Health and Substance Use The University of Sydney Sydney Australia

**Keywords:** adolescent health, alcohol, E‐cigarette use, modifiable behaviours, poor diet, smoking, sociodemographic disadvantage

## Abstract

**Introduction:**

Recent Australian adolescent data on the prevalence of chronic disease risk behaviours among diverse sociodemographic groups is lacking. This study examined the prevalence of dietary intake (sugar‐sweetened beverages (SSBs); discretionary foods; fruit; vegetables), and alcohol (standard drink; binge drinking), tobacco, and e‐cigarette use, across adolescents of diverse socioeconomic status (SES) and geographical locations.

**Methods:**

Cross‐sectional data were analysed from 4445 adolescents across 71 schools in 2022 as part of the 36‐month follow‐up survey from the school‐based cluster randomised controlled trial, *Health4Life* (M_age_ = 15.7 years, SD = 0.6; 47.0% female‐identifying). Fourteen percent (*n* = 571) were categorised as low SES and 86% (*n* = 3518) as mid‐to‐high SES, relative to the study sample, with 9% (*n* = 399) from regional areas. Binary logistic regressions compared differences for each outcome across SES and geographical locations, controlling for gender, psychological distress, intervention status, and school clustering.

**Results:**

Low SES adolescents had a lower prevalence of excessive discretionary food intake (PR = 0.87, 95% CI = 0.77–0.99), standard drink consumption (PR = 0.78; 95% CI = 0.65–0.93) and binge drinking (PR = 0.68; 95% CI = 0.50–0.92) compared to mid‐to‐high SES adolescents. Regional adolescents had a higher prevalence of standard drink consumption (PR = 1.41; 95% CI = 1.00–1.97), binge drinking (PR = 1.77; 95% CI = 1.07–2.93), and tobacco smoking (PR = 2.06; 95% CI = 1.18–3.60) compared to adolescents in major cities. Excessive discretionary food intake was less prevalent among adolescents from disadvantaged backgrounds (PR = 0.84, 95% CI = 0.76–0.94) compared to more advantaged adolescents.

**Conclusion:**

Chronic disease risk behaviours among adolescents differ across SES and geographical locations, with regional adolescents fairing considerably worse across alcohol and tobacco use outcomes. Prevention for diet‐related behaviours should be improved for more advantaged adolescents, while tailored interventions to SES and geographical location separately may be required for alcohol‐, tobacco‐, and e‐cigarette use.

**So What?:**

Public health policy and interventions targeting chronic disease risk behaviours must prioritise the needs of low SES and regional adolescents to reduce health inequities.

**Trial Registration:**

The Health4Life trial is registered with the Australian New Zealand Clinical Trials Registry (ACTRN12619000431123)

## 1 Background

Chronic disease risk behaviours including poor diet, alcohol use, and tobacco smoking are well‐known contributors to chronic diseases such as cancer, diabetes, and cardiovascular and lung diseases [1]. In Australia, these are among the most prevalent chronic diseases and contribute significantly to the national burden of disease [2]. Chronic diseases are the leading cause of mortality in Australia [3] and accounted for 85% of total disease burden in 2023 [4]. Compounding this issue, individuals living in lower socioeconomic areas and areas outside of major cities experience a greater prevalence of chronic conditions compared to their counterparts in higher socioeconomic areas and major cities, respectively [4]. These health disparities—and restricted opportunities for cultivating healthy lifestyles—may in part be influenced by factors such as stigma [5], limited social support [6], restricted employment opportunities, limited infrastructure, and greater geographic spread, impacting access to health care options [7]. This sociodemographic gradient in health is persistent across varying life stages (e.g., early life disadvantage can influence health risk in adulthood [8] and health and educational challenges [9]). Thus, preventing known chronic disease risk behaviours is a key step towards reducing health inequities for lower socioeconomic status (SES) and/or ‘regional’, ‘rural’, and ‘remote’ populations (herein referred to as ‘disadvantaged’). Recent research has also highlighted growing concerns regarding the health risks and harmful effects associated with e‐cigarette use (vaping), with adolescents especially vulnerable [10, 11]. A study of 5114 Australian adolescents found that the risk of initiating tobacco smoking was almost five times higher for those who had ever used an e‐cigarette compared to those who had not, with younger adolescents reporting a considerably greater risk [12]. As many of these chronic disease risk behaviours emerge during adolescence, prevention efforts during this period that facilitate establishing healthy lifestyle habits protective against chronic disease are key for promoting long‐term health outcomes [13, 14, 15]. However, to ensure health‐promoting education and prevention efforts benefit *all* adolescents, it is first important to understand the prevalence and patterns of chronic disease risk behaviours among adolescents from disadvantaged backgrounds compared to their more advantaged counterparts.

Previous national data and studies suggest that disadvantaged adolescents are generally more likely to engage in chronic disease risk behaviours, such as not meeting national dietary guidelines and consuming unhealthy foods [16, 17], and reporting higher rates of risky alcohol consumption and tobacco smoking [16, 18, 19], compared to their more advantaged peers. International research demonstrates similar findings [20, 21, 22]. Vaping prevalence trends, however, appear to vary between Australia and other countries. In Australia, among people aged 14 and over, those with high SES report a higher likelihood of vaping (6.6%) compared to those of low SES (3.1%) [23] although other research has reported no difference by SES in vaping prevalence among Australian adolescents [24]. International studies, however (e.g., United Kingdom, United States, and Germany) have reported a greater prevalence of vaping among adolescents of lower SES and/or from regional areas compared to their counterparts [25, 26, 27]. Nevertheless, much of this evidence provides a snapshot of these diet‐related, alcohol, tobacco and e‐cigarette use risk behaviours assessed separately from each other across different study samples. Additionally, limited recent research has explored how these behaviours collectively differ between disadvantaged (low SES and/or regional‐based) and more advantaged adolescents in Australia. Although Champion et al. [28] and Gardner et al. [24] examined these behaviours in the same Health4Life Study sample and found no sociodemographic differences, neither study assessed these behaviours collectively across disadvantaged groups (i.e., combining low SES and/or regional for comparison to more advantaged adolescents), nor did they adjust for gender and psychological distress which can be associated with these behaviours [29, 30, 31]. Additionally, Champion et al.'s [28] analysis focused on 11–14‐year‐olds, thus before typical initiation ages for alcohol and tobacco use among Australian adolescents [32, 33].

The current study addresses shortcomings in the existing literature by using data from a large, geographically diverse, Australian cluster randomised controlled trial (RCT) to better understand differences in chronic disease risk behaviours between disadvantaged and more advantaged adolescent groups in Australia, while controlling for gender and psychological distress. Specifically, this study aims to: (1) examine the prevalence of poor dietary intake (excessive sugar‐sweetened beverages (SSBs) and discretionary foods, and insufficient fruit and vegetables), alcohol use, tobacco smoking, and vaping among adolescents of low SES versus mid‐to‐high SES, and between regional and metropolitan adolescents and; (2) compare the prevalence of these behaviours among disadvantaged (low SES and/or living regionally) versus more advantaged (mid‐to‐high SES and/or living in metropolitan areas) adolescents.

## 2 Methods

The methods for this study have been written per the Strengthening the Reporting of Observational Studies in Epidemiology (STROBE) guidelines for cross‐sectional studies [34].

### 2.1 Data Source

The current study uses 36‐month follow‐up data from the *Health4Life* Study collected between 1 July and 31 December 2022. The full study methodology, recruitment and consent procedures for *Health4Life* are reported elsewhere [35, 36]. Briefly, *Health4Life* is a large cluster RCT that commenced in 2019 and involved 6639 Year 7 (baseline age 11–14 years) students from 71 secondary schools across three states in Australia (New South Wales [NSW], Queensland [QLD] and Western Australia [WA]). The study sample included approximately 51% independent, 30% government, and 19% Catholic schools, with 90% of students located in major cities and 10% in inner or outer regional areas (see Appendix 1 for baseline sociodemographic details). The baseline sample was comparable to the Australian 11–14 year old population in terms of sex and Australian‐born status; however, it was more advantaged [37]. It also differed by school type and location from the 2022 national enrolment profile, where 64% of all secondary students attended government schools, 20% attended Catholic schools, and 16% attended independent schools, with 60% in major cities and 40% outside of major cities [38]. Participation was voluntary, with school‐level consent provided by principals. Of the 71 schools, 40 used opt‐out parental consent, and 31 required active written and oral parental consent. All students gave active written consent. The intervention was delivered during Year 7 health education classes and consisted of six co‐designed 20‐min interactive cartoon storylines imparting evidence‐based information about poor diet, alcohol use, tobacco smoking, vaping, physical inactivity, screen time, and poor sleep [39]. It was supported by optional teacher‐facilitated activities, factsheets, quizzes, and personalised feedback. Control schools delivered health education as usual. Over the study period, data were collected by students completing self‐report online surveys during class, with a total of 4445 participants (67%) having completed the 36‐month follow‐up survey, constituting the sample for this study (see Appendix 2 for the CONSORT diagram). Ethical approvals for the study were received from the University of Sydney (2018/882), the University of Queensland (2019000037), Curtin University (HRE2019–0083), and relevant school sector ethics committees.

## 3 Measures

### 3.1 Demographic Characteristics

SES, geographical location, gender identity and symptoms of psychological distress were self‐reported by students. Participants' SES was measured using the Family Affluence III (FASIII) scale at baseline [40, 41]. As children and adolescents may be unaware of their parents' income, education, and other socioeconomic indicators, the FASIII provides a practical approach for assessing family affluence by asking about material assets in the home including: car ownership (response options: no, one, two or more); having their own bedroom (response options: no, yes); computer ownership (response options: none, one, two, three or more); number of bathrooms (response options: none, one, two, three or more); dishwasher ownership (response options: no, yes); and overseas family holidays in the previous year (response options: not at all, once, twice, more than twice). The summed FASIII scores were transformed into ridit scores, which ranked the relative SES within the study sample on a continuous scale between 0 (lowest SES) and 1 (highest SES). The ridit scores were then used to categorise participants into lower, middle and upper SES groups relative to the study population [42]. Thus participants were considered as relatively low, mid, or high SES. Participants' school location was used as a proxy for their geographical location as many students were unsure of their home postcode. Geographical location of schools were measured using the Australian Statistical Geography Standard Remoteness Structure [43], which classifies geographical areas as major city, inner regional, outer regional, remote, or very remote, with remoteness increasing as distance from services increases. Gender identity was measured using an Australian Bureau of Statistics‐recommended gender‐related indicator research question that asked participants to describe their gender including responses for ‘male’, ‘female’, ‘non‐binary’, ‘other identity’ or ‘prefer not to answer’ [44].

### 3.2 Psychological Distress

The presence of psychological distress symptoms were measured using the Kessler six‐item scale (K6) which has been validated among adolescents and measures the frequency of symptoms such as feeling nervous, hopeless, restless, worthless in the previous month [45, 46]. Participants responded to questions on a 5‐point Likert scale with answers scored from “none of the time” to “all of the time”. Higher K6 scores indicated greater psychological distress. K6 scores were dichotomised based on published cut‐points for serious mental illness (SMI), where scores above 13 indicated a SMI (=1 Yes) and those below 13 suggested no SMI (=0 No) [47].

### 3.3 Chronic Disease Risk Behaviours

#### 3.3.1 Diet

Students self‐reported their consumption of SSBs, fruit, vegetables, and discretionary foods in response to questions from the Student Physical Activity and Nutrition Survey (SPANS) [48]. To assess SSB consumption, participants were asked about how many cups of water, fruit juice, diet soft drinks (e.g., Diet Coke) and soft drinks (e.g., Coke), cordials or sports drinks they usually consumed. A figure showing different sizes of these drinks was shown to facilitate student responses (see Appendices pg. 5). Due to the lack of universally adopted cut‐offs for “excessive” SSB or discretionary food consumption, thresholds were informed via consensus with nutrition and health behaviour experts, and national health recommendations. As consuming 5+ cups of SSBs/week is one of the highest response options used in SPANS, the current study dichotomised SSB consumption with 5+ cups/week coded as excessive (=1) and less than 5 cups as not (=0). For discretionary food consumption, participants reported how often they usually consumed junk foods such as hot chips, snack foods such as cakes, confectionery items (e.g., chocolate), ice cream, and takeaway meals from fast food outlets such as McDonald's (see Appendices pg. 6). The Australian Dietary Guidelines recommend limiting or avoiding these foods [49]; therefore, this variable was dichotomised, where consuming discretionary food items once or more per day was coded as excessive (=1) and responses less than this as not excessive (=0). For fruit and vegetables, participants were asked how many servings of fruit and vegetables they usually eat each day. A pictorial chart showing serving sizes of fruit and vegetables was shown to assist participant responses (see Appendices pg. 7). Fruit and vegetable variables were separately dichotomised to indicate < 2 servings of fruit/day or < 5 servings of vegetables/day as insufficient fruit or vegetable intake (=1) and 2+ servings of fruit/day or 5+ servings of vegetables/day as sufficient (=0), in line with the Australian National Dietary Guidelines [49].

#### 3.3.2 Alcohol

A single‐item assessed participants' consumption of a full standard drink in the last 6 months, “Have you had a full standard alcoholic drink in the past 6 months?” (0 = No, 1 = Yes). Binge‐drinking in the previous 6 months was assessed via “How often did you have 5 or more standard alcoholic drinks on one occasion in the past 6 months”. Participants answering ‘never’ were categorised as did not binge drink (=0), and any responding between ‘less than monthly’ to ‘daily or almost daily’ were categorised as did binge drink in the previous 6 months (=1). A visual aid showing standard drink sizes of various alcoholic beverages according to the Australian Drinking Guidelines was shown alongside alcohol‐related questions to facilitate participants' responses (see Appendices pg. 8–10).

#### 3.3.3 Tobacco

Previous 6‐month tobacco use was captured using a single item measure from the Youth Risk Behaviour Survey, with participants asked, “In the past 6 months, have you tried cigarette smoking, even one or two puffs?” (0 = No, 1 = Yes) [50].

#### 3.3.4 Vaping

Previous 6‐month vape use was assessed using two items. First, participants were asked, “Have you ever used an e‐cigarette, even one or two puffs?”. Participants who responded “Yes” were then asked, “When did you last use e‐cigarettes?”. Responses ranging between, “In the past day (24‐hours)” to “More than 3‐months ago, but within the past 6‐months” were categorised as “Yes = 1”. Responses ranging from “More than 6 months ago, but within the past 12‐months” to “More than 12‐months ago” were categorised as “No = 0”, as were those who answered “No” to having ever used an e‐cigarette.

## 4 Statistical Analysis

Descriptive statistics summarised sample characteristics and prevalence estimates of each chronic disease risk behaviour across three groupings. Group 1 compared adolescents of low SES to mid‐to‐high SES. Group 2 compared adolescents in regional areas to those in major cities. Group 3 compared disadvantaged adolescents (i.e., low SES and/or living regionally) to more advantaged adolescents (i.e., mid or high SES and/or living in major cities). Binary logistic regression models using the prLogistic package in R calculated prevalence ratios (PRs) for each outcome following the conditional standardisation procedure, with 95% confidence intervals (CIs) estimated using the delta method [51]. Three separate models were run per outcome. The first model compared mid‐to‐high SES to low SES, adjusting for gender identity, psychological distress, intervention status (i.e., *Health4Life* vs. Control group), school‐level clustering and geographical location. The second model focused on geographical location (major city versus regional), with gender identity, psychological distress, intervention status, school‐level clustering and SES (mid‐to‐high SES versus low SES) as covariates. The third model compared disadvantaged (i.e., low SES and/or living regionally) to more advantaged participants (i.e., mid or high SES and/or living in major cities), adjusting only for gender identity, psychological distress, intervention status and school‐level clustering. A significant difference in prevalence was reported if the 95% CI did not cross 1. Complete case analysis was used for each model, the missing data for each outcome ranged from 4.0% to 5.4% and the sample size was considerable; therefore, no additional analysis of missing data was done [52]. The only exception was that 11% of student responses to the discretionary food consumption measure were missing; however, results from a simple regression analysis indicated missing responses were not related to greater discretionary food consumption risk at baseline. Further details of the simple regression analysis are reported in Appendix 6, pg. 16. Data analysis was done using R version 4.3.3.

### 4.1 Results

**FIGURE 1:**
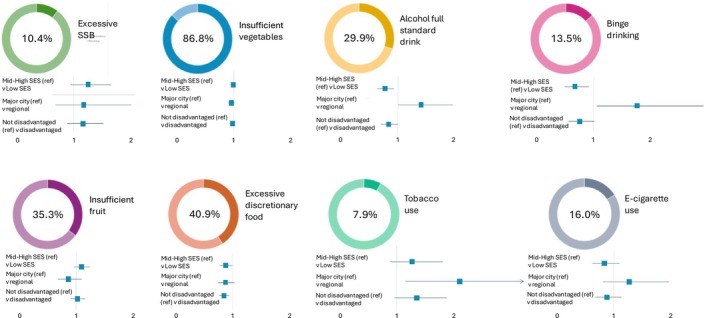
Prevalence of risk behaviours among 4445 participants aged 14–17 years in the Health4Life trial at 36‐month post‐baseline follow‐up, and prevalence ratios (with 95% confidence intervals), by socio‐demographic characteristics*.The forest plots per outcome represent three separate models, all of which adjust for gender, psychological distress, intervention status and school clustering. Model 1 compared mid‐to‐high SES versus low SES, additionally adjusting for geographical location. Model 2 compared major city versus regional, also adjusting for SES. Model 3 compared more advantaged (mid‐to‐high SES and/or major city) versus disadvantaged (low SES and/or regional). Reference groups are denoted in brackets. *The data for this figure are in Appendix 11, pg. 21–22.

Among the 4445 participants (M_age_ = 15.7 years, SD = 0.6; 47.0% female‐identifying), 54% (*n* = 2398) were located in NSW, 27.3% (*n* = 1212) in QLD and 18.8% (*n* = 835) in WA, with 91% attending schools in major city areas (remaining 9% in regional areas). Fourteen percent (*n* = 571) of participants were classified as low SES and 86% (*n* = 3518) as mid‐to‐high SES relative to the study sample. Further details of the sample characteristics are reported in Table 1 (see appendices 7 and 9, pg. 17–19, for sample characteristics grouped by relative SES and geographical location, respectively). The overall prevalence of risk behaviours among the study sample is included in Figure 1.

**TABLE 1:** Selected sample characteristics of participants (*N* = 4445).

	Whole sample(*N* = 4445)	Disadvantaged sub‐sample(*N* = 896)	More advantagedsub‐sample (*N* = 3549)
Age (mean [SD])	15.71 (0.64)	15.71 (0.66)	15.69 (0.53)
Gender identity (*N* [%])			
Male	2172 (49.0%)	516 (57.7%)	1656 (46.8%)
Female	2065 (46.6%)	336 (37.6%)	1729 (48.9%)
Non‐binary	123 (2.8%)	25 (2.8%)	98 (2.8%)
Prefer not to say	73 (1.7%)	17 (1.9%)	56 (1.6%)
Psychological distress (*N* [%])			
No	3349 (79.9%)	674 (81.0%)	2675 (79.6%)
Yes	844 (20.1%)	158 (19.0%)	686 (20.4%)
State (*N* [%])			
NSW	2398 (54.0%)	520 (58.0%)	1878 (52.9%)
QLD	1212 (27.3%)	127 (14.2%)	1085 (30.6%)
WA	835 (18.8%)	249 (27.8%)	586 (16.5%)
Socio‐economic status (*N* [%])			
Low	571 (14%)	571 (66.9%)	0 (0%)
Mid‐to‐High	3518 (86.0%)	283 (33.1%)	3235 (100%)
Geographical location (*N* [%])			
Major city	4046 (91%)	497 (55.5%)	3549 (100%)
Regional	399 (9%)	399 (44.5%)	0 (0%)

### 4.2 Model 1: SES (Low SES Versus Mid‐To‐High SES) Comparison

Compared to participants from mid‐to‐high SES backgrounds, excessive discretionary food consumption was 13% less prevalent among low SES participants (PR = 0.87; 95% CI = 0.77–0.99). Low SES participants also reported a 22% lower prevalence of consuming a full standard drink (PR = 0.78; 95% CI = 0.65–0.93) and a 32% lower prevalence of binge drinking (PR = 0.68; 95% CI = 0.50–0.92) in the previous 6 months (Figure 1; Appendices 8 and 11, pg. 18–22). No significant differences in the prevalence of consuming SSBs, fruit, and vegetables, tobacco smoking, and vaping were reported between participants of low SES backgrounds compared to mid‐to‐high SES backgrounds.

### 4.3 Model 2: Geographical Location (Regional Versus Major City) Comparison

Compared to participants from major cities, the participants from regional areas reported a 41% higher prevalence of consuming a full standard drink (PR = 1.41; 95% CI = 1.00–1.97), 77% higher prevalence of binge drinking (PR = 1.77; 95% CI = 1.07–2.93), and a twofold increase in the prevalence of tobacco smoking (PR = 2.06; 95% CI = 1.18–3.60) in the previous 6 months (Figure 1; Appendices 10 and 11, pg. 20–22). No significant differences in the prevalence of vaping, or consuming SSBs, fruit, vegetables, and discretionary foods, were reported between participants from regional areas and major cities.

### 4.4 Model 3: Disadvantaged (Low SES and/or Regionally Located) Compared to More Advantaged (Mid‐To‐High SES and/or Major City)

After adjusting for gender, psychological distress, intervention status, and school clustering, only excessive discretionary food consumption (PR = 0.84; 95% CI = 0.76–0.94) was significantly less prevalent (16%) among disadvantaged compared to more advantaged adolescents (Figure 1; Appendices 6 and 11, pg. 16–22). All other behaviours did not significantly differ in prevalence between disadvantaged compared to more advantaged participants.

## 5 Discussion

This cross‐sectional study investigated differences in the prevalence of eight chronic disease risk behaviours by sociodemographic and geographical location in a large sample of Australian adolescents aged 14–17 years. We found that adolescents of lower SES compared to those classified as mid‐to‐high SES reported a lower prevalence of excessive discretionary food intake (13%), consuming a full standard alcoholic drink (22%), and binge drinking (32%). Contrastingly, geographical location differences did not follow this trend. Regional participants reported a higher prevalence of consuming a full standard drink (41%) and binge drinking (77%) than participants in major cities. Alarmingly, regional participants had a more than twofold increase in tobacco smoking prevalence compared to their peers in major cities, highlighting a significant public health concern. When comparing disadvantaged adolescents (low SES and/or regional) to more advantaged (mid‐to‐high SES and/or major city) adolescents, only discretionary food intake was significantly lower (16%) among disadvantaged adolescents.

Our findings that excessive discretionary food consumption was lower among disadvantaged adolescents compared to more advantaged adolescents, and among adolescents of lower SES compared to those of mid‐to‐high SES, suggest low SES may uniquely protect against excessive discretionary food consumption. This contrasts with previous Australian research showing greater fast‐food consumption among low SES adolescents than their more advantaged peers [53, 54], and among those in metropolitan areas compared to rural areas [54]. It may be that social (e.g., cultural‐related food practices), environmental (e.g., local food banks) and economic (e.g., cost of fast‐food proportionate to income) factors potentially prevent low SES adolescents from consuming excessive discretionary foods. Although some research suggests price discounting and promotions are more common on discretionary foods than healthier foods in Australia [55, 56]. Nevertheless, low‐income households generally spend less on meals out and fast food than high‐income households [57], reducing opportunities to eat discretionary foods. This is potentially related to the high and rising costs of goods in Australia including take‐away foods, which can take up a large proportion of their disposable income [58, 59]. Moreover, disadvantaged populations are more likely to rely on food assistance programmes which tend to provide healthier food items [60]. This highlights the importance of public health efforts addressing environmental and economic factors influencing dietary choices across diverse sociodemographic adolescent populations. Disadvantaged adolescents remain a key priority due to the unique barriers to health and generally greater clustering of chronic disease risk behaviours they experience. However, these findings suggest there may also be value in complementary strategies to support more advantaged adolescents to reduce their intake of discretionary foods, if further research supports these results.

The alcohol‐related outcomes demonstrate an interesting contrast. While consuming a full standard drink and binge drinking were less prevalent among adolescents of lower SES compared to mid‐to‐high SES, the opposite was seen for regional adolescents who had a higher prevalence of these outcomes than adolescents in major cities. This suggests alcohol patterns are uniquely different between low SES and regional adolescents. These findings align with recent wastewater analysis from various metropolitan, regional, and socioeconomic areas across Australia showing greater alcohol consumption in higher compared to lower socioeconomic areas, and select regional areas compared to major cities [61]. For those classified as lower SES adolescents in our study, the cost of alcohol may have been a barrier, or social, cultural, and familial factors could have played a role, particularly if they identified as culturally and linguistically diverse which can be protective against alcohol consumption [62, 63]. On the other hand, regional adolescents may have been more likely to obtain alcohol from parents and experience less parental disapproval towards alcohol use compared to their peers in major cities, thus encouraging higher alcohol consumption [18, 64].

Aligning with previous literature, tobacco smoking was more prevalent among regional adolescents versus those based in major cities [19]. This is especially concerning, as tobacco smoking is not only associated with immediate health consequences (e.g., shortness of breath) and heightened risk for chronic disease, but rural adolescents who have tried cigarettes at least once may be more likely than their counterparts to become a daily smoker [65]. Considering the high potential for tobacco dependence after as few as four tobacco cigarettes and the challenges of quitting without support–even when there is a desire to–this further adds to regional adolescents' vulnerability to chronic disease burden as they have less access to smoking cessation and other treatment services [66, 67, 68]. This disparity in smoking prevalence may stem from greater exposure to friends or parents who smoke, which are known risk factors [69, 70], particularly as regional households with children tend to have a greater proportion of smokers compared to major city households [71].

In terms of vaping, although there were no significant differences across all comparisons in our study, we typically saw a lower prevalence of vaping among lower SES adolescents compared to mid‐to‐high SES adolescents, and higher prevalence in regional compared to major city‐based adolescents, consistent with previous Australian data [12, 23]. This may reflect greater affluence among 79.3% of regional adolescents (categorised as mid‐to‐high SES relative to study sample; see Appendix 9, pg. 19) making e‐cigarettes more affordable. Other risk factors include tobacco use, family or friends tobacco use, low‐risk perception of vaping, and exposure to marketing about e‐cigarettes or tobacco cigarettes [72]. Despite strict regulations governing the marketing of e‐cigarettes in Australia, e‐cigarette marketing is highly visible on social media [73], where vendors exploit loopholes to discreetly market and sell e‐cigarettes [74]. These factors may have been more overt among regional adolescents, particularly as we saw a higher prevalence of tobacco smoking among regional adolescents than in major cities. Protective factors, however, include perceived cost and dangers of vaping, parental monitoring, not knowing or seeing someone who used e‐cigarettes [72]. For low SES adolescents, some of these factors, such as the cost of e‐cigarettes coupled with less exposure to vaping marketing [75] may have contributed to the lower vaping prevalence than mid‐to‐high SES peers.

Our findings point to the nuanced differences in the prevalence of these chronic disease risk behaviours across SES and geographical location. They suggest that targeted strategies may be required to target diet behaviours among more affluent adolescents. In contrast, alcohol, tobacco smoking, and vaping behaviours may require tailoring to SES and geographical location separately. Public health policy and interventions must appropriately address the needs of diverse sociodemographic adolescent populations, with particular focus on disadvantaged adolescents who are vulnerable to experiencing health inequity [4]. These prevention efforts should adopt a holistic approach integrating early intervention, health promotion (e.g., accessible health information), and policy change (e.g., stronger partnerships with low SES and regional communities to implement tailored evidence‐based prevention programs). Ensuring equitable coverage is essential; digital interventions targeting these behaviours can be effective [76] and should be considered for large‐scale prevention, particularly for low SES and regional adolescents as they have promise in reaching broad and diverse populations with less access barriers.

## 6 Strengths and Limitations

The use of self‐reported measures in the current study is prone to bias as participants may have under‐ or over‐reported aligning with social desirability of responses, although objective measures were not feasible considering the size of this RCT. As only 67% of the original cohort completed the 36‐month follow‐up, this attrition rate may have impacted the generalisability of findings and introduced potential bias if those lost to follow‐up systematically differed from those retained. Nevertheless, the study maintains a large sample size, with a follow‐up rate comparable to other longitudinal studies. The study sample was predominantly from major cities and of relative mid‐to‐high SES, indicating many disadvantaged adolescents were not included. Given that government schools tend to enrol a higher proportion of students of lower SES [77], and the current study only included 34% of government schools, this may partially explain the skew towards greater affluence. In addition, no population weighting was applied, which may limit the generalisability of the prevalence estimates to the broader Australian student population. Nevertheless, the current study still included a large and relatively diverse cohort of > 4000 students completing the survey from independent, Catholic and government schools across NSW, WA, and QLD. Future research examining the current prevalence of these behaviours and a more representative sample is needed. It could also consider the multiple layers of disadvantage and intersectionality of various factors on adolescent chronic disease risk behaviours, such as sole parent status, LGBTQI+ identity, mental health status, language background and other marginalised identities.

## 7 Conclusion

The current study has filled a gap in the literature regarding the prevalence of poor diet (insufficient fruit and vegetables, and excessive SSBs and discretionary food consumption), alcohol use (consumption of full standard drink and binge drinking), tobacco use and vaping between disadvantaged and more advantaged adolescent groups in Australia. Adolescents of lower SES reported less alcohol consumption, binge drinking, and excessive food consumption compared to adolescents of mid‐to‐high SES, potentially influenced by social, cultural and economic factors including cost of alcohol and discretionary foods. Contrastingly, adolescents in regional areas compared to major cities had higher prevalences of alcohol consumption, binge drinking, and tobacco smoking suggesting current prevention approaches are not adequately reaching regional adolescents. Tracking these behaviours over time and examining representative samples to gain accurate prevalence estimates is crucial for informing prevention efforts to target these behaviours prior to them solidifying in the adolescents' behavioural repertoire. To safeguard the future health of all adolescents, prevention efforts should adopt a holistic approach integrating early intervention, health promotion, and policy change tailored proportionately to the needs of diverse adolescent groups. The findings of this study strongly reinforce the need for proportionate and targeted prevention approaches, given the variability of poor diet, alcohol, tobacco and vaping behaviours reported across diverse sociodemographic adolescent groups.

## Ethics Statement

The Health4Life Study was conducted in accordance with the ethical standard outlined in the 1964 Declaration of Helsinki and approved by Human Research Ethics Committees of the University of Sydney (2018/882), the University of Queensland (2019000037), Curtin University (HRE2019–0083), and relevant school sector ethics committees.

## Conflicts of Interest

The authors declare no conflicts of interest.

## Supporting information


**Data S1.** Supporting information.

## Data Availability

The data that support the findings of this study are available on request from the corresponding author. The data are not publicly available due to privacy or ethical restrictions.
