# Supplementary material for: Who Reports Greater Chronic Disease Risk Behaviours? A Closer Look at Sociodemographic Differences Among Australian Adolescents: A Cross‐Sectional Analysis
Source: Health Promot J Austr. 2025 Jul 15;36(4):e70079. doi: 10.1002/hpja.70079 (PMC12263350; doi:10.1002/hpja.70079)
Supplement: Supplementary file 1 — Data S1. Supporting information. [file HPJA-36-0-s001.docx]

Appendices

Table of Contents

[Appendix 1. Baseline sociodemographic characteristics of the 6639 Year 7 students aged 11-14 years who participated in the *Health4Life* study 2](#_Toc200900509)

[Appendix 2. CONSORT Diagram 3](#_Toc200900510)

[Appendix 3. Question asked to measure participants’ gender-identity 4](#_Toc200900511)

[Appendix 4. Primary outcomes survey instruments 5](#_Toc200900512)

[Diet-related variables survey questions 5](#_Toc200900513)

[Alcohol-related variables survey questions 8](#_Toc200900514)

[Tobacco-related variables survey questions 11](#_Toc200900515)

[Vaping-related variables survey questions 12](#_Toc200900516)

[Appendix 5: STROBE cross-sectional reporting checklist 14](#_Toc200900517)

[Appendix 6: Risk behaviours among participants present at 36-month post-baseline follow-up in the Health4Life trial, grouped by disadvantaged versus more advantaged participants 16](#_Toc200900518)

[Appendix 7: Sample characteristics among participants present at 36-month post-baseline follow-up in the Health4Life trial, grouped by relative Low SES versus Mid to High SES levels 17](#_Toc200900519)

[Appendix 8: Risk behaviours among participants present at 36-month post-baseline follow-up in the Health4Life trial, grouped by relative Low SES versus Mid to High SES levels 18](#_Toc200900520)

[Appendix 9: Sample characteristics of participants present at 36-month post-baseline follow-up in the Health4Life trial, grouped by geographical location 19](#_Toc200900521)

[Appendix 10: Risk behaviours among participants present at 36-month post-baseline follow-up in the Health4Life trial, grouped by geographical location 20](#_Toc200900522)

[Appendix 11: Prevalence ratios with 95% confidence intervals (CI) for each risk behaviour among 4445 participants present at 36-month post-baseline follow-up in the Health4Life trial 21](#_Toc200900523)

[Forest plots for each primary outcome 23](#_Toc200900524)

[Appendix 12: Excessive SSB consumption forest plot 23](#_Toc200900525)

[Appendix 13: Insufficient fruit intake forest plot 24](#_Toc200900526)

[Appendix 14: Insufficient vegetable intake forest plot 25](#_Toc200900527)

[Appendix 15: Excessive discretionary food consumption forest plot 26](#_Toc200900528)

[Appendix 16: Consumed full standard drink of alcohol in previous 6-months forest plot 27](#_Toc200900529)

[Appendix 14: Binge drank alcohol in previous 6-months forest plot 28](#_Toc200900530)

[Appendix 17: Tobacco smoking in previous 6-months forest plot 28](#_Toc200900531)

[Appendix 18: E-cigarette use in previous 6-months forest plot 29](#_Toc200900532)

[**References** 30](#_Toc200900533)

# Appendix 1. Selected baseline sociodemographic characteristics of the 6639 Year 7 students aged 11-14 years who participated in the *Health4Life* study (1)

|  | **Health4Life sample** | |
| --- | --- | --- |
|  | **Students** | **Proportion** |
| State |  |  |
| New South Wales (*N* = 37) | 3535 | 53.2% (39.2–67.3%) |
| Queensland (*N* = 18) | 1789 | 26.9% (14.3–39.6%) |
| Western Australia (*N* = 16) | 1315 | 19.8% (9.7–29.9%) |
| School type |  |  |
| Government (*N* = 24) | 2003 | 30.1% (17.2–42.9%) |
| Independent (*N* = 37) | 3377 | 50.9% (36.7–65.1%) |
| Catholic (*N* = 10) | 1259 | 19.0% (6.6–31.3%) |
| Remoteness |  |  |
| Major city | 5953 | 89.7% (82.8–96.5%) |
| Inner or outer regional | 686 | 10.3% (3.5–17.2%) |
| Socio-economic status |  |  |
| Lower | 909 | 15.1% (12.6–17.6%) |
| Middle | 2209 | 36.7% (34.3–39.1%) |
| Higher | 2896 | 48.2% (44.2–52.1%) |
| No response/missing data | 625 | — |

# Appendix 2. CONSORT Diagram

**Excluded** (n= 434 schools)

♦  Did not meet inclusion criteria or declined to participate

Enrolment

Approached to participate (n= 519 schools)

**Analysed 3 Year Follow-Up**(n= 36 schools), (n= 2,394 Year 10 students)

**Analysed** **3 Year Follow-Up**(n= 35 schools), (n= 2,051 Year 10 students)

**3 Year Follow-Up**

2 Year Follow-Up

**3 Year Follow-Up**
(n= 35 schools), (n= 2,051 Year 10 students) (67.7%)

Not assessed: 979 students (32.3%; absent, insufficient data, moved schools, declined)

**3 Year Follow-Up**(n= 36 schools), (n= 2,394 Year 10 students) (66.3%)

Not assessed: 1216 students (33.7%; absent, insufficient data, moved schools, declined)

2 Year Follow-Up
(n= 36 schools), (n= 2,648 Year 9 students) (74.4%)

Not assessed: 962 students (26.6%; absent, insufficient data, moved schools, declined)

2 Year Follow-Up
(n= 35 schools), (n= 2,367 Year 9 students) (78.1%)

Not assessed: 663 students (21.9%; absent, insufficient data, moved schools, declined)

1 Year Follow-Up

(n= 36 schools), (n= 2,949 Year 8 students) (81.7%)

Not assessed: 661 students (18.3%; absent, insufficient data, moved schools, declined)

1 Year Follow-Up

1 Year Follow-Up

(n= 35 schools), (n= 2,577 Year 8 students) (85%)

Not assessed: 453 students (15.0%; absent, insufficient data, moved schools, declined)

Post-intervention Follow-Up

(n= 36 schools), (n= 3,034 Year 7 students) (84%)

Not assessed: 576 students (16.0%; absent, insufficient data, moved schools, declined)

Post-intervention Follow-Up

(n= 35 schools), (n= 2,664 Year 7 students) (88%)

Not assessed: 366 students (12.0%; absent, insufficient data, moved schools, declined)

Post-intervention
Follow-Up

Baseline Survey

(n= 35 schools), (n= 3,030 Year 7 students)

Not assessed: 391 students (11.43%; absent, insufficient data, student did not consent)

Baseline

Baseline Survey

(n= 36 schools), (n= 3,610 Year 7 students)

Not assessed: 133 students (3.55%; absent, insufficient data, student did not consent)

Allocation

**Allocated to control**(n= 43 schools*), (n= 4,704 Year 7 students)

Students with parental consent: 3,421 (72.73%)

*7 schools withdrew before baseline due to a lack of time. 1 school withdrew post-baseline and is not included in the final baseline sample.

**Allocated to Health4Life intervention**
(n= 42 schools*), (n= 4,576 Year 7 students)

Students with parental consent: 3,743 (81.80%)

*5 schools withdrew before baseline due to a lack of time. 1 school withdrew after baseline is not included in the final baseline sample.

**Randomised**
(n= 85 schools),
(n= 9,280 Year 7 students)

# Appendix 3. Question asked to measure participants’ gender-identity


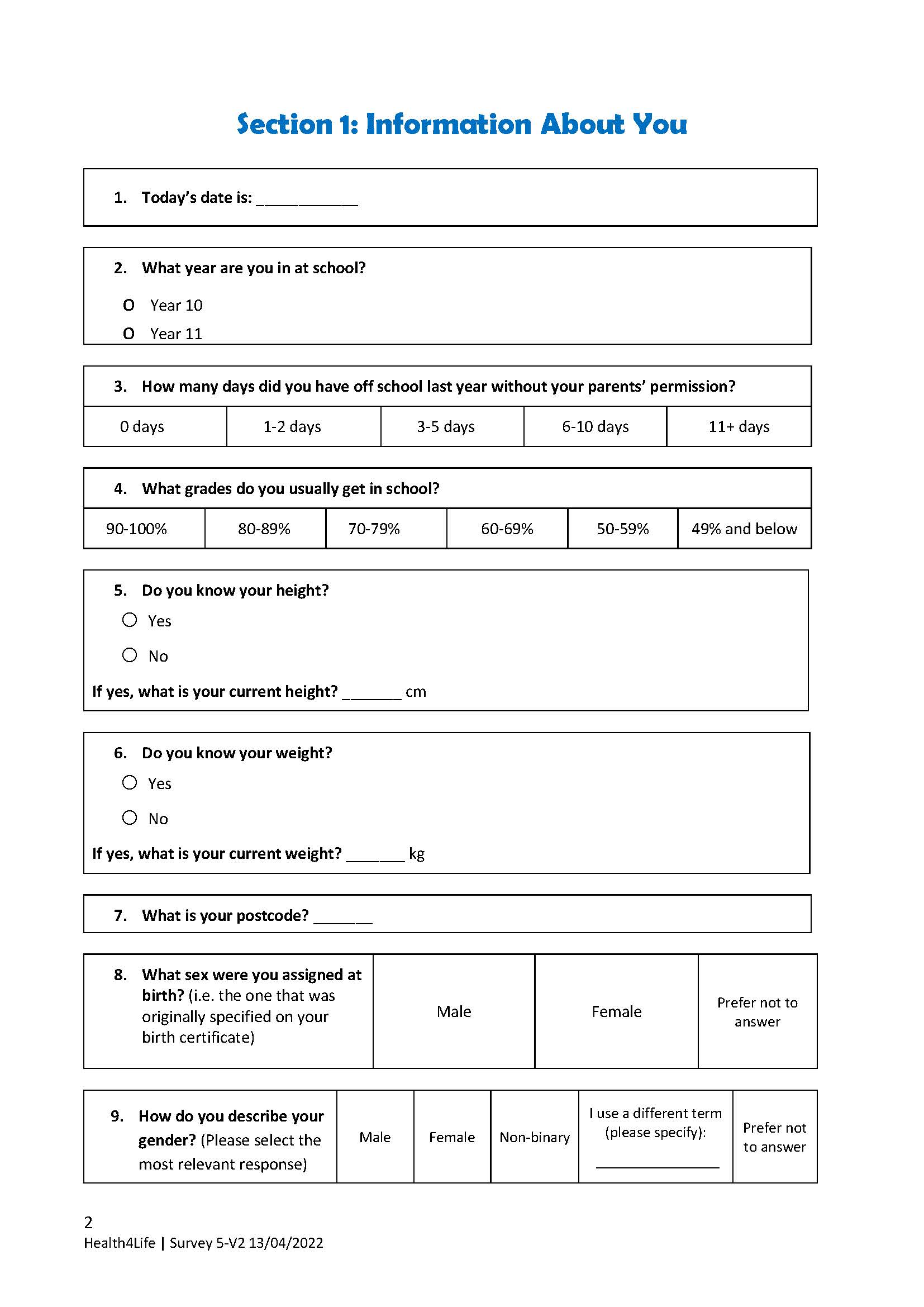


# Appendix 4. Primary outcomes survey instruments

Diet-related variables survey questions
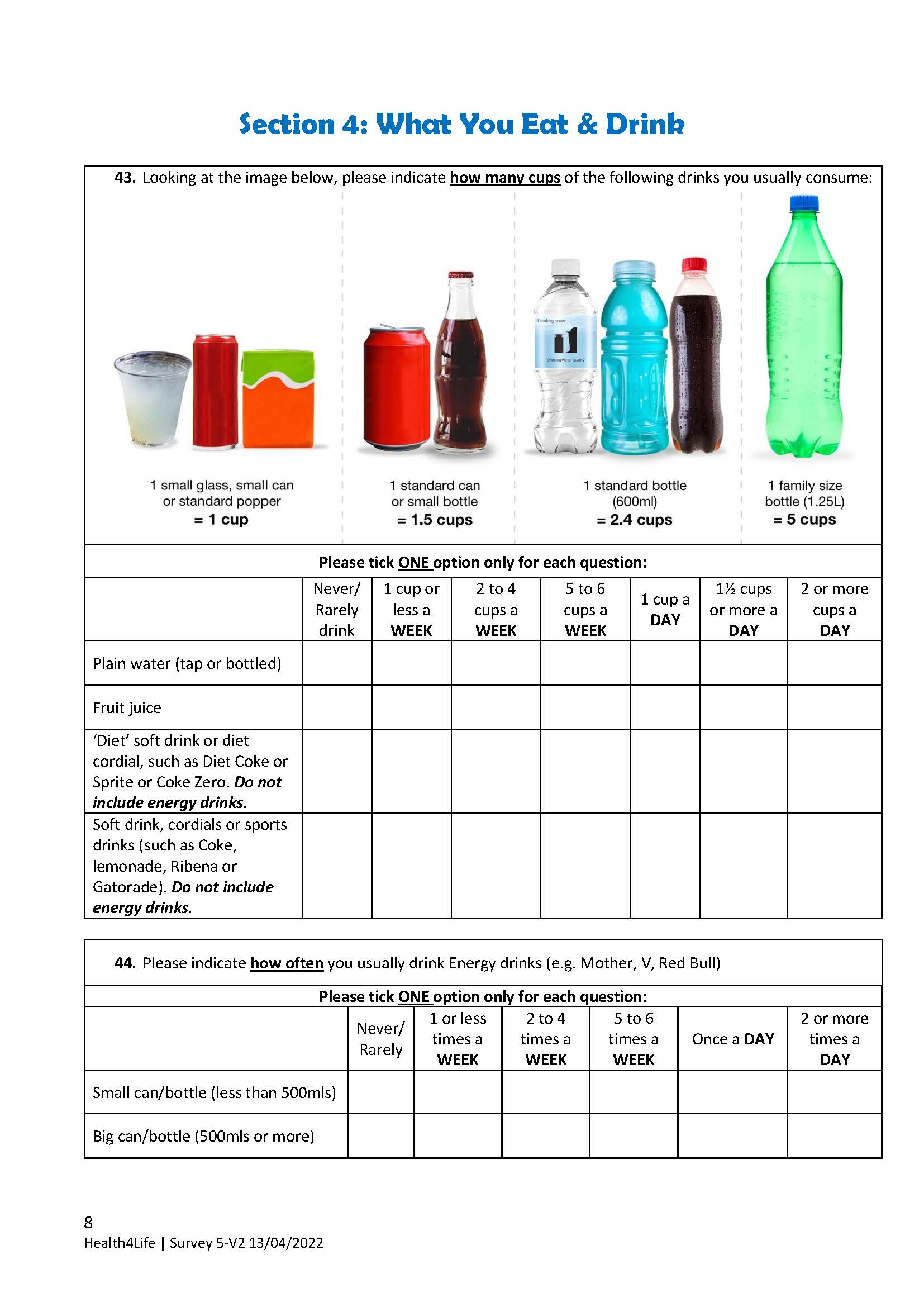


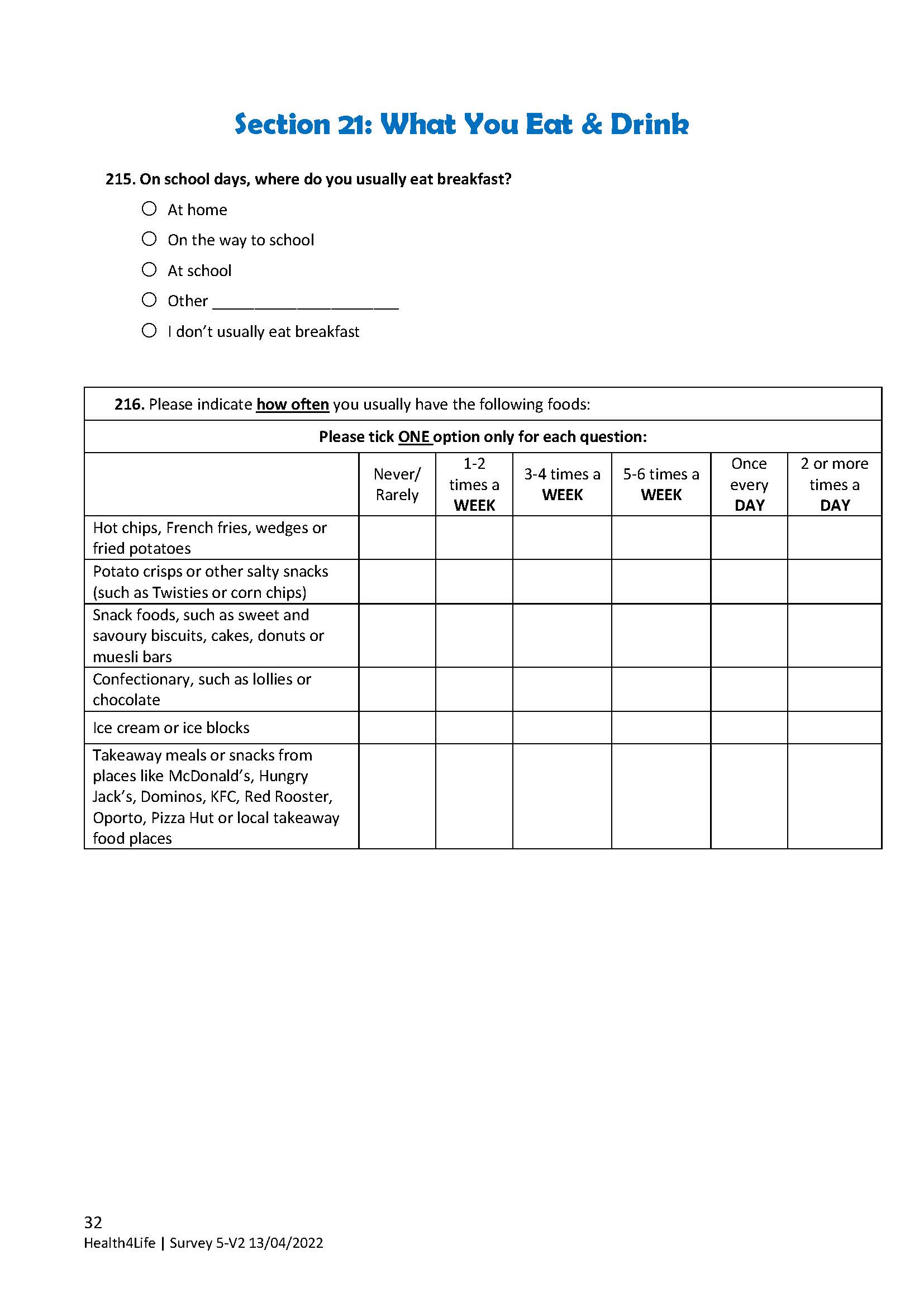


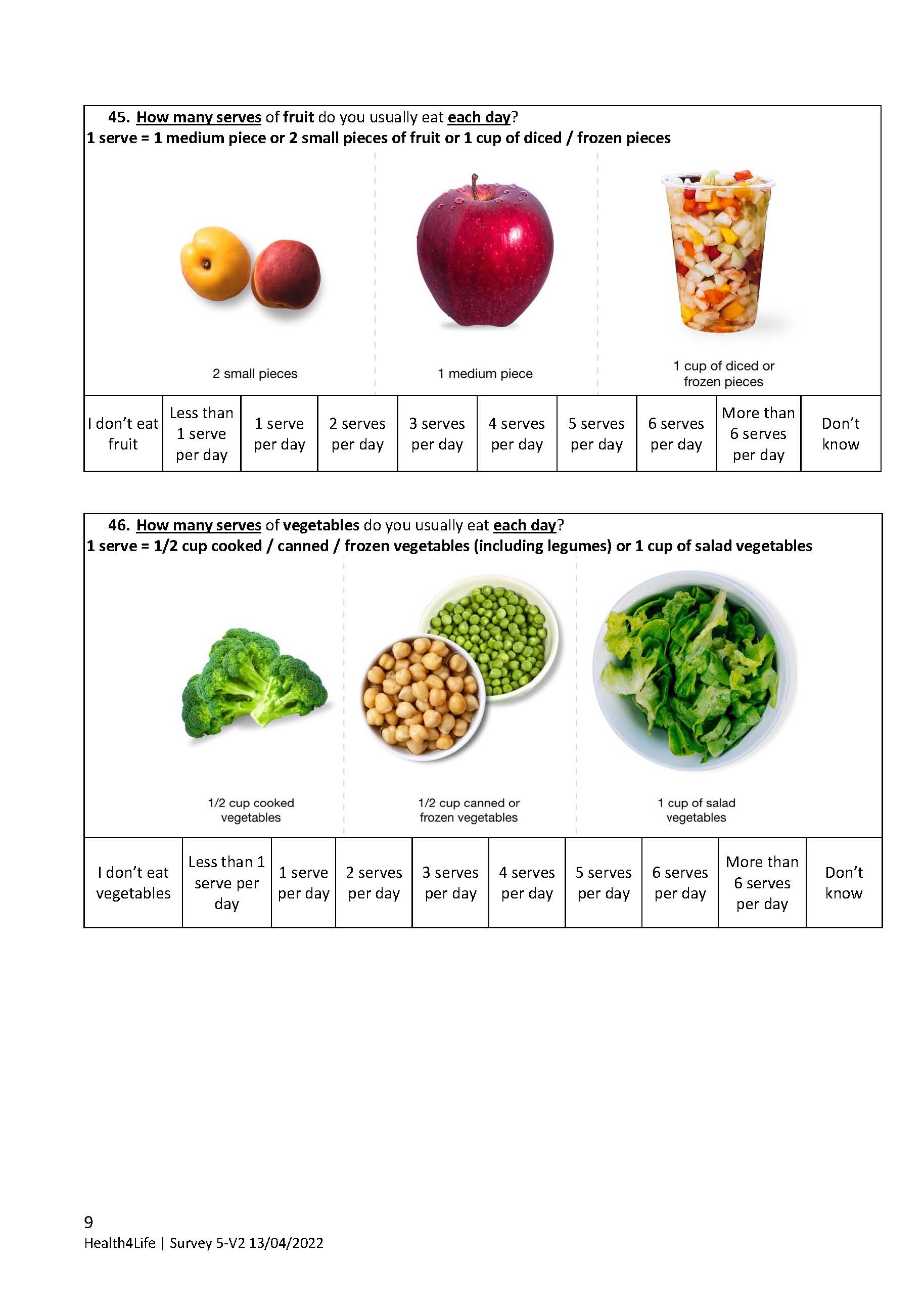


## Alcohol-related variables survey questions


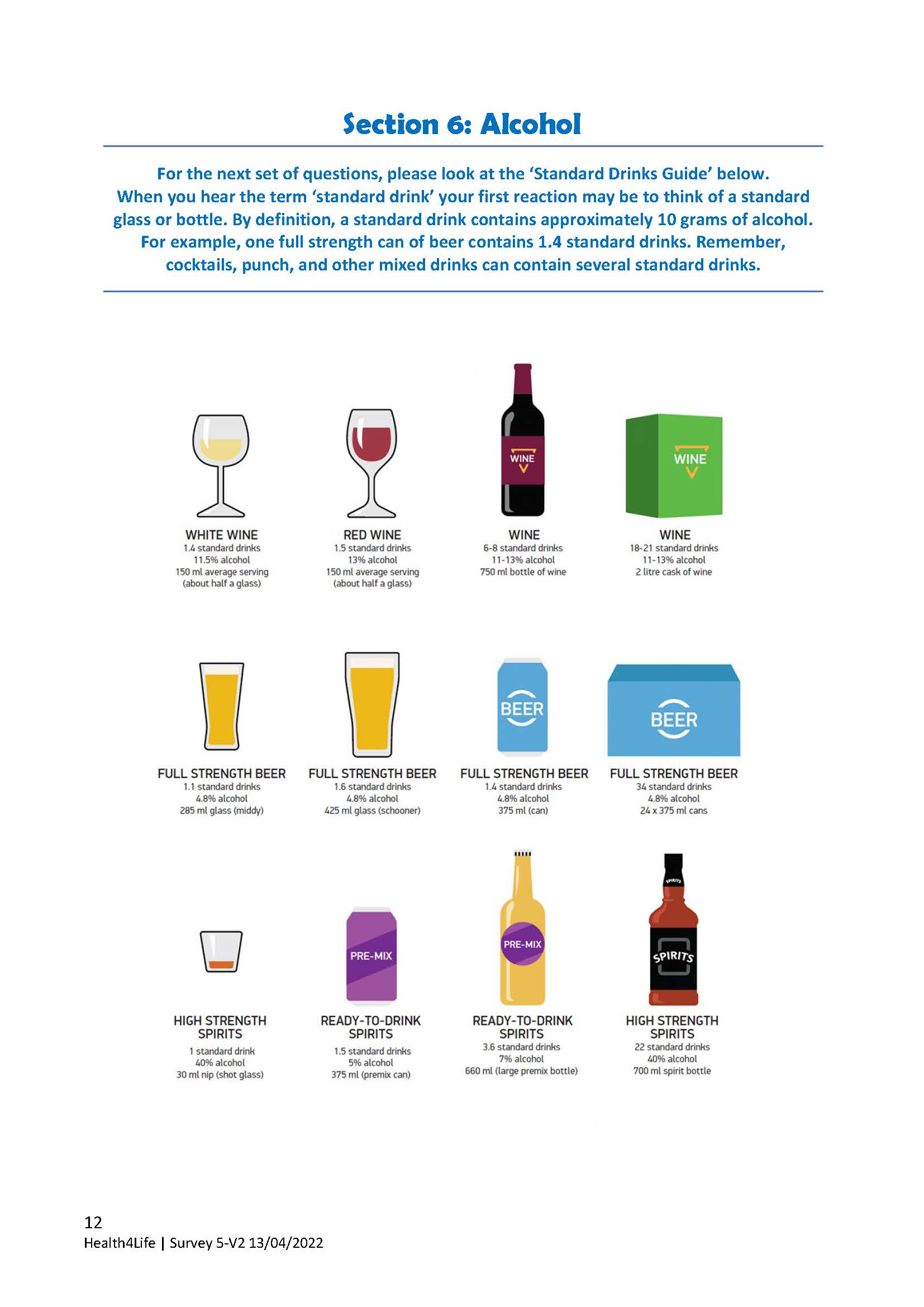


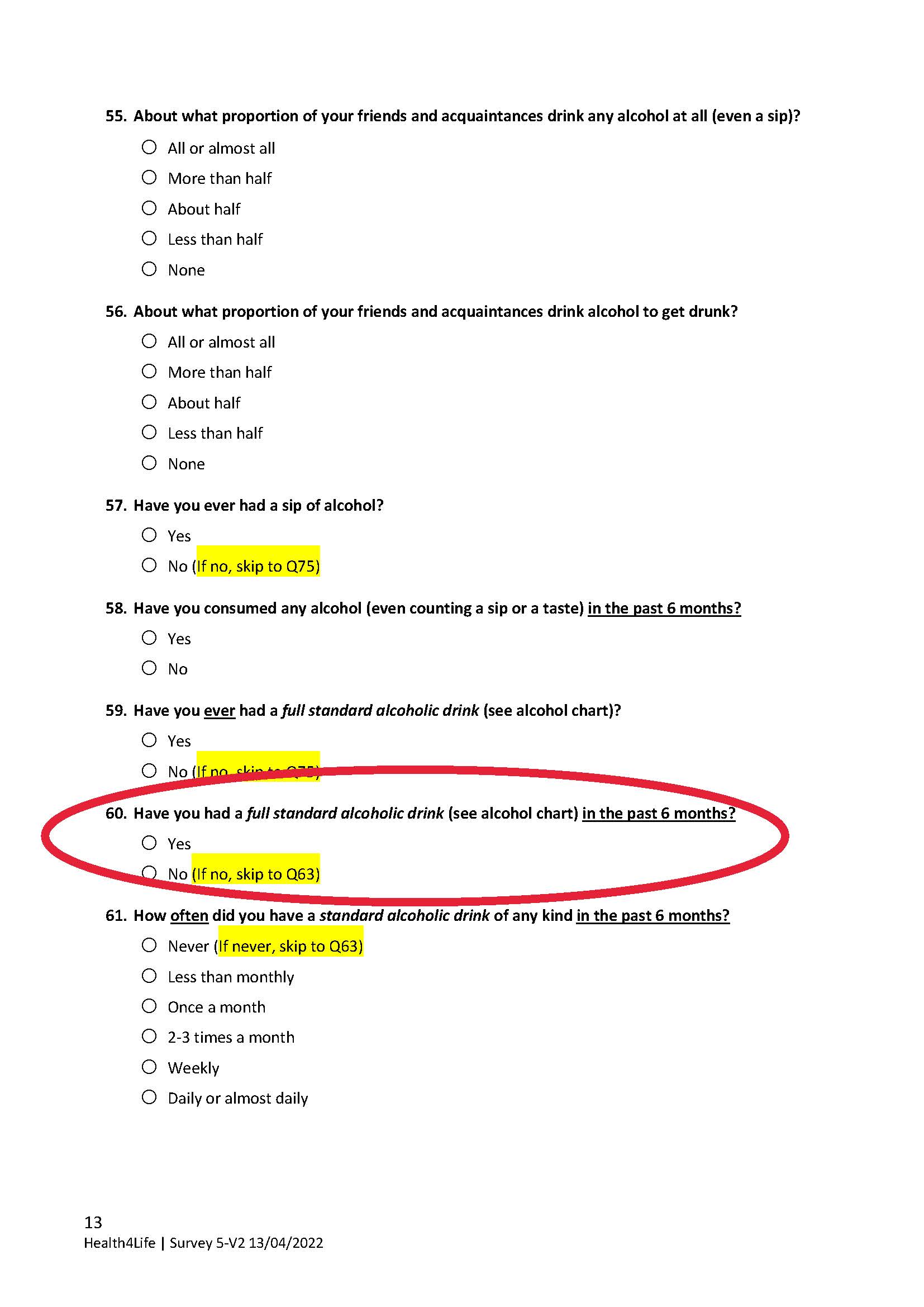


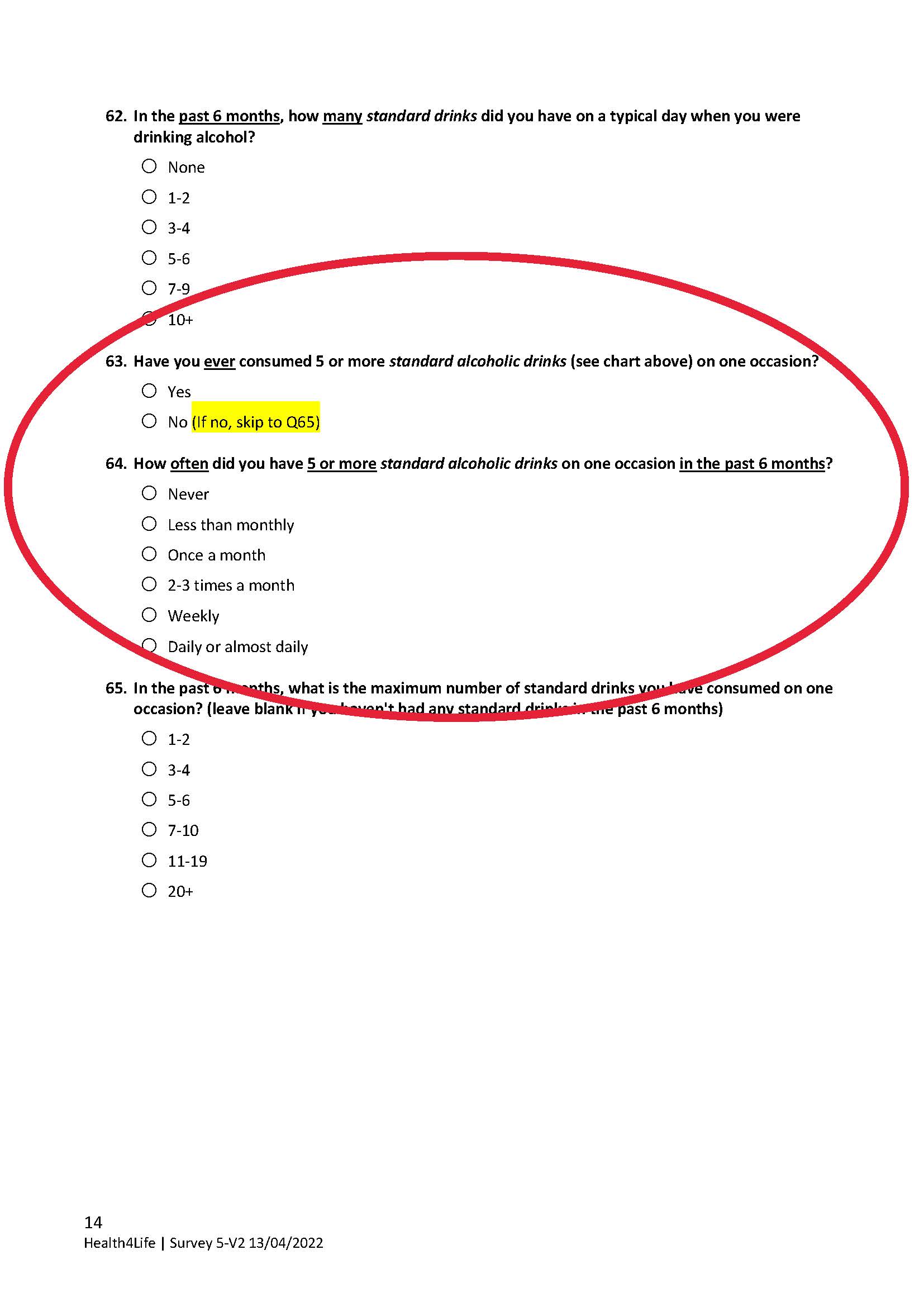


## Tobacco-related variables survey questions


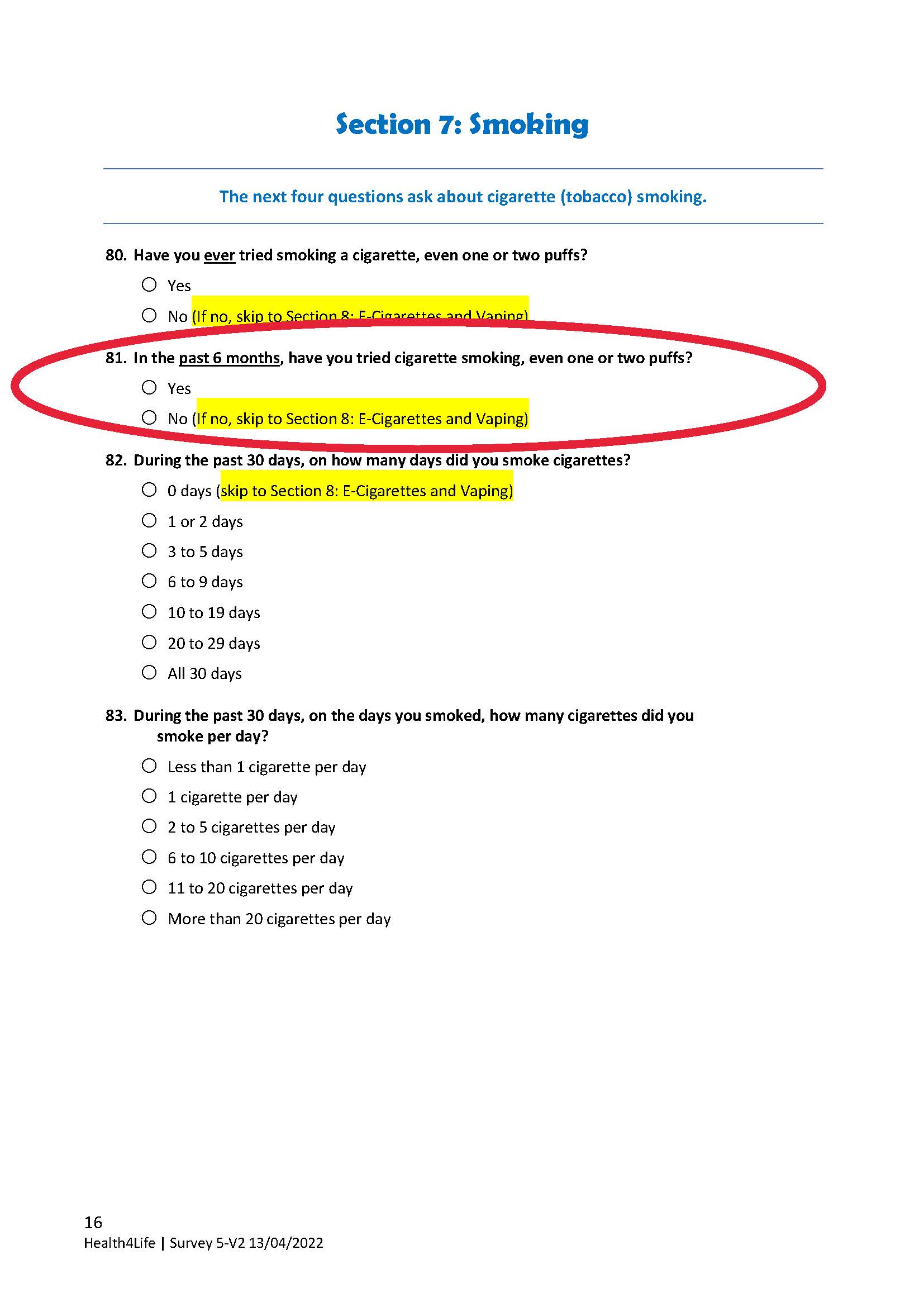


## Vaping-related variables survey questions


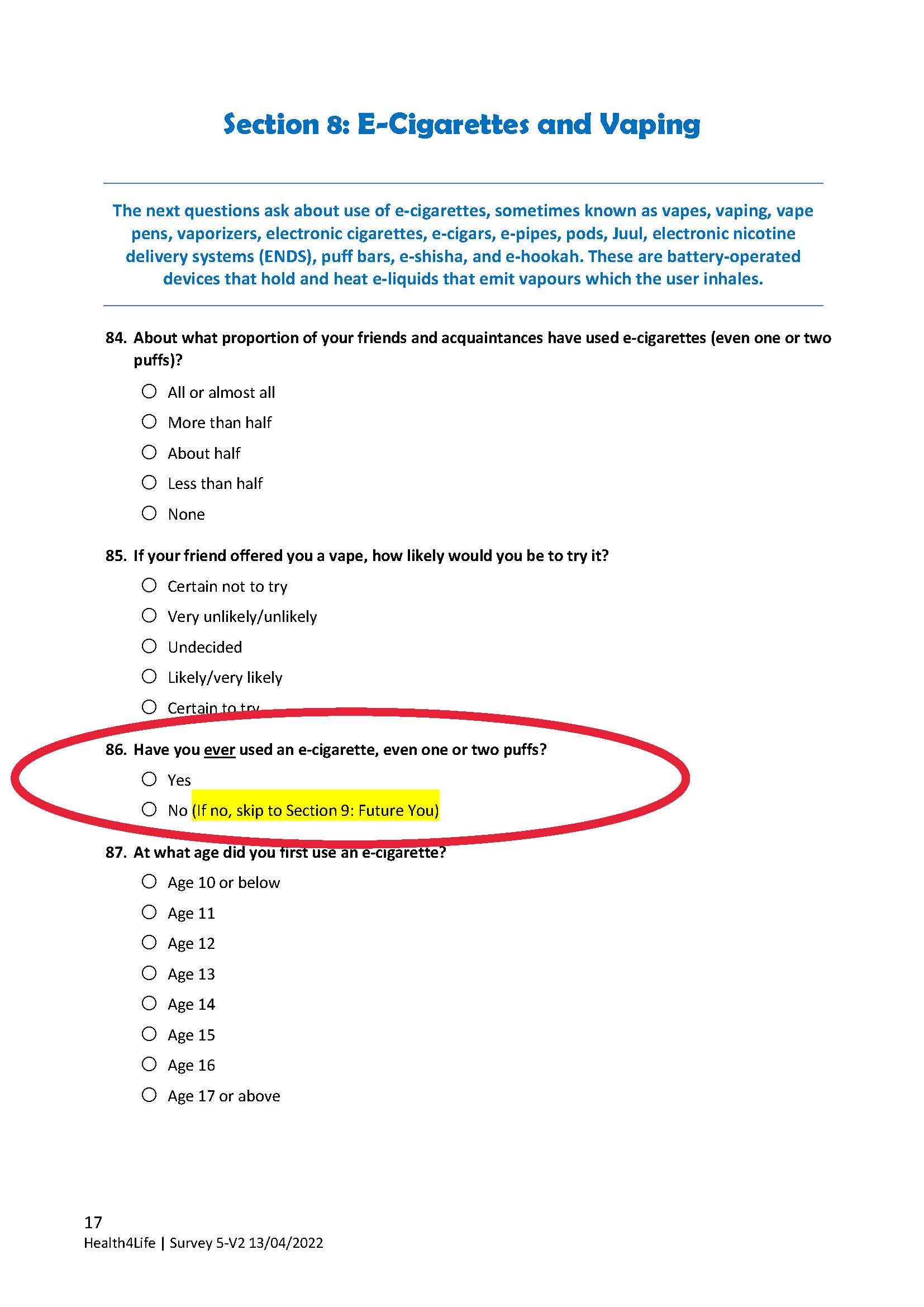


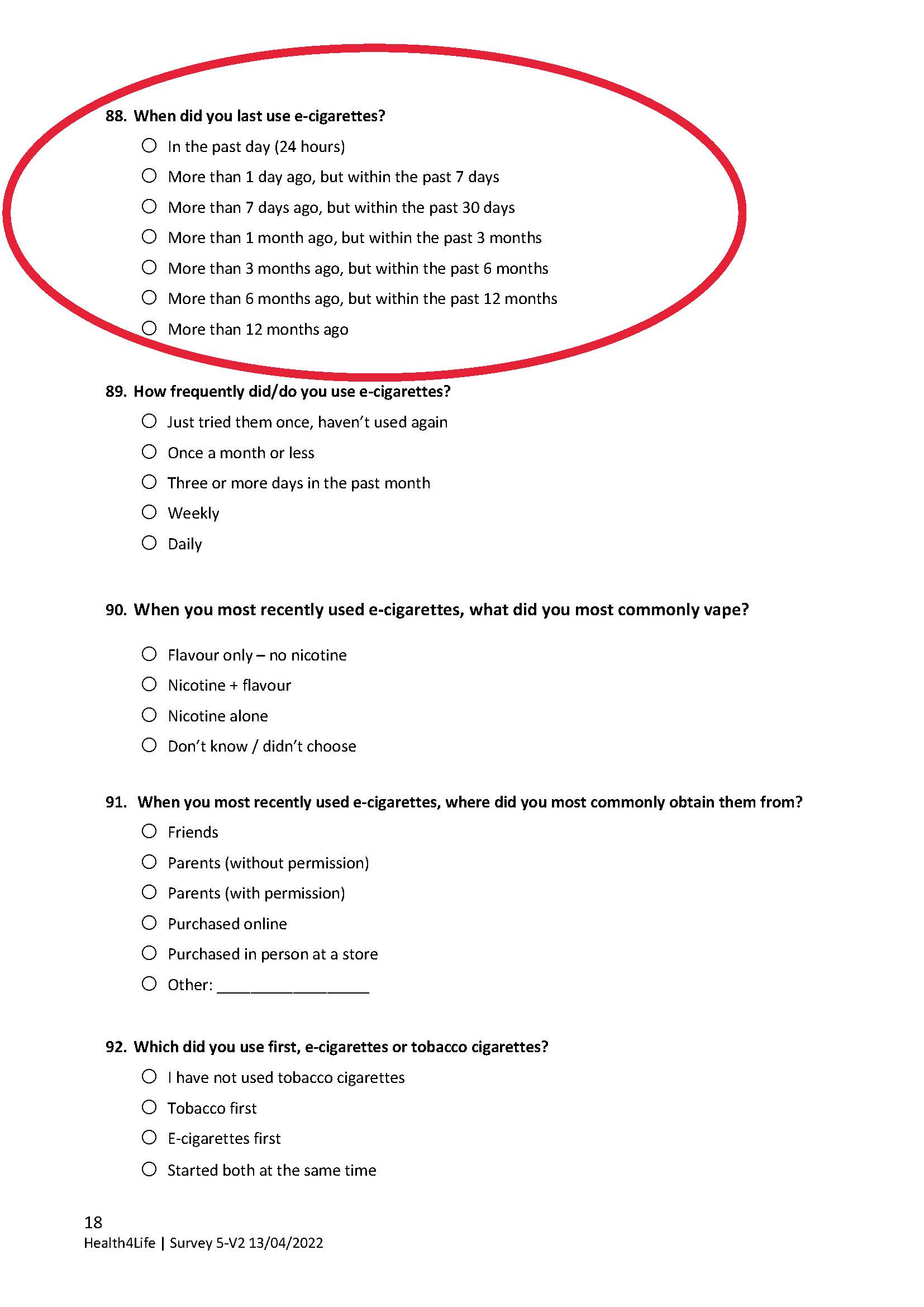


# Appendix 5: STROBE cross-sectional reporting checklist

|  |  | Reporting Item | Page Number |
| --- | --- | --- | --- |
| Title and abstract |  |  |  |
| Title | [#1a](https://www.goodreports.org/reporting-checklists/strobe-cross-sectional/info/#1a) | Indicate the study’s design with a commonly used term in the title or the abstract |  |
| Abstract | [#1b](https://www.goodreports.org/reporting-checklists/strobe-cross-sectional/info/#1b) | Provide in the abstract an informative and balanced summary of what was done and what was found |  |
| Introduction |  |  |  |
| Background / rationale | [#2](https://www.goodreports.org/reporting-checklists/strobe-cross-sectional/info/#2) | Explain the scientific background and rationale for the investigation being reported | 4-6 |
| Objectives | [#3](https://www.goodreports.org/reporting-checklists/strobe-cross-sectional/info/#3) | State specific objectives, including any prespecified hypotheses | 7 |
| Methods |  |  |  |
| Study design | [#4](https://www.goodreports.org/reporting-checklists/strobe-cross-sectional/info/#4) | Present key elements of study design early in the paper | 7 |
| Setting | [#5](https://www.goodreports.org/reporting-checklists/strobe-cross-sectional/info/#5) | Describe the setting, locations, and relevant dates, including periods of recruitment, exposure, follow-up, and data collection | 8 |
| Eligibility criteria | [#6a](https://www.goodreports.org/reporting-checklists/strobe-cross-sectional/info/#6a) | Give the eligibility criteria, and the sources and methods of selection of participants. | N/A |
|  | [#7](https://www.goodreports.org/reporting-checklists/strobe-cross-sectional/info/#7) | Clearly define all outcomes, exposures, predictors, potential confounders, and effect modifiers. Give diagnostic criteria, if applicable | 8-12 |
| Data sources / measurement | [#8](https://www.goodreports.org/reporting-checklists/strobe-cross-sectional/info/#8) | For each variable of interest give sources of data and details of methods of assessment (measurement). Describe comparability of assessment methods if there is more than one group. Give information separately for exposed and unexposed groups if applicable. | 8-12 |
| Bias | [#9](https://www.goodreports.org/reporting-checklists/strobe-cross-sectional/info/#9) | Describe any efforts to address potential sources of bias | N/A |
| Study size | [#10](https://www.goodreports.org/reporting-checklists/strobe-cross-sectional/info/#10) | Explain how the study size was arrived at | 8 |
| Quantitative variables | [#11](https://www.goodreports.org/reporting-checklists/strobe-cross-sectional/info/#11) | Explain how quantitative variables were handled in the analyses. If applicable, describe which groupings were chosen, and why | 12 |
| Statistical methods | [#12a](https://www.goodreports.org/reporting-checklists/strobe-cross-sectional/info/#12a) | Describe all statistical methods, including those used to control for confounding | 12 |
| Statistical methods | [#12b](https://www.goodreports.org/reporting-checklists/strobe-cross-sectional/info/#12b) | Describe any methods used to examine subgroups and interactions | N/A |
| Statistical methods | [#12c](https://www.goodreports.org/reporting-checklists/strobe-cross-sectional/info/#12c) | Explain how missing data were addressed | 8 |
| Statistical methods | [#12d](https://www.goodreports.org/reporting-checklists/strobe-cross-sectional/info/#12d) | If applicable, describe analytical methods taking account of sampling strategy | 12 |
| Statistical methods | [#12e](https://www.goodreports.org/reporting-checklists/strobe-cross-sectional/info/#12e) | Describe any sensitivity analyses | N/A |
| Results |  |  |  |
| Participants | [#13a](https://www.goodreports.org/reporting-checklists/strobe-cross-sectional/info/#13a) | Report numbers of individuals at each stage of study—eg numbers potentially eligible, examined for eligibility, confirmed eligible, included in the study, completing follow-up, and analysed. Give information separately for for exposed and unexposed groups if applicable. | N/A |
| Participants | [#13b](https://www.goodreports.org/reporting-checklists/strobe-cross-sectional/info/#13b) | Give reasons for non-participation at each stage | N/A |
| Participants | [#13c](https://www.goodreports.org/reporting-checklists/strobe-cross-sectional/info/#13c) | Consider use of a flow diagram | N/A |
| Descriptive data | [#14a](https://www.goodreports.org/reporting-checklists/strobe-cross-sectional/info/#14a) | Give characteristics of study participants (eg demographic, clinical, social) and information on exposures and potential confounders. Give information separately for exposed and unexposed groups if applicable. | 8 |
| Descriptive data | [#14b](https://www.goodreports.org/reporting-checklists/strobe-cross-sectional/info/#14b) | Indicate number of participants with missing data for each variable of interest | - |
| Outcome data | [#15](https://www.goodreports.org/reporting-checklists/strobe-cross-sectional/info/#15) | Report numbers of outcome events or summary measures. Give information separately for exposed and unexposed groups if applicable. | N/A |
| Main results | [#16a](https://www.goodreports.org/reporting-checklists/strobe-cross-sectional/info/#16a) | Give unadjusted estimates and, if applicable, confounder-adjusted estimates and their precision (eg, 95% confidence interval). Make clear which confounders were adjusted for and why they were included | 13-16 |
| Main results | [#16b](https://www.goodreports.org/reporting-checklists/strobe-cross-sectional/info/#16b) | Report category boundaries when continuous variables were categorized | - |
| Main results | [#16c](https://www.goodreports.org/reporting-checklists/strobe-cross-sectional/info/#16c) | If relevant, consider translating estimates of relative risk into absolute risk for a meaningful time period | N/A |
| Other analyses | [#17](https://www.goodreports.org/reporting-checklists/strobe-cross-sectional/info/#17) | Report other analyses done—e.g., analyses of subgroups and interactions, and sensitivity analyses | N/A |
| Discussion |  |  |  |
| Key results | [#18](https://www.goodreports.org/reporting-checklists/strobe-cross-sectional/info/#18) | Summarise key results with reference to study objectives | 17 |
| Limitations | [#19](https://www.goodreports.org/reporting-checklists/strobe-cross-sectional/info/#19) | Discuss limitations of the study, taking into account sources of potential bias or imprecision. Discuss both direction and magnitude of any potential bias. | 20 |
| Interpretation | [#20](https://www.goodreports.org/reporting-checklists/strobe-cross-sectional/info/#20) | Give a cautious overall interpretation considering objectives, limitations, multiplicity of analyses, results from similar studies, and other relevant evidence. | 17-20 |
| Generalisability | [#21](https://www.goodreports.org/reporting-checklists/strobe-cross-sectional/info/#21) | Discuss the generalisability (external validity) of the study results | 20 |
| Other Information |  |  |  |
| Funding | [#22](https://www.goodreports.org/reporting-checklists/strobe-cross-sectional/info/#22) | Give the source of funding and the role of the funders for the present study and, if applicable, for the original study on which the present article is based | 22 |

None The STROBE checklist is distributed under the terms of the Creative Commons Attribution License CC-BY.

# Appendix 6: Risk behaviours among participants present at 36-month post-baseline follow-up in the Health4Life trial, grouped by disadvantaged versus more advantaged participants (unadjusted values)

| Risk behaviour | Whole sample (N = 4445) | Disadvantaged sub-sample (N = 896) | More advantaged sub-sample (N = 3549) |
| --- | --- | --- | --- |
| **SSB consumption** | | | |
| More than 5 cups (N [% [95% CI]]) | 444/4272  (10.4% [9.5,11.3]) | 112/853  (13.1% [11.0,15.6]) | 332/3419  (9.7% [8.8,10.7]) |
| **Fruit intake** | | | |
| Insufficient fruit (N [% [95% CI]]) | 1508/4273  (35.3% [33.9,36.7]) | 309/854  (36.2% [33.0,39.5]) | 1199/3419  (35.1% [33.5,36.7]) |
| **Vegetable intake** | | | |
| Insufficient vegetable (N [% [95% CI]]) | 3708/4272  (86.8% [85.7,87.8]) | 719/854  (84.2% [81.6,86.5]) | 2989/3419  (87.4% [86.3,88.5]) |
| **Discretionary food intake^[[1]](#footnote-1)^** | | | |
| Excessive (N [% [95% CI]]) | 1621/3960  (40.9% [39.4,42.5]) | 279/775  (36.0% [32.7,39.4]) | 1342/3185  (42.1% [40.4,43.9]) |
| **Full standard drink in previous 6 months** | | | |
| Did consume full standard drink | 1263/4225  (29.9% [28.5,31.3]) | 251/844  (29.7% [26.8,32.9]) | 1012/3381  (29.9% [28.4,31.5]) |
| **Binge drink in previous 6 months** | | | |
| Did binge drink | 569/4207  (13.5% [12.5,14.6]) | 115/842  (13.7% [11.5,16.1]) | 454/3365  (13.5% [12.4,14.7]) |
| **Tobacco smoking in previous 6 months** | | | |
| Did smoke tobacco cigarette | 334/4212  (7.9% [7.2,8.8]) | 86/834  (10.3% [8.4,12.6]) | 248/3378  (7.3% [6.5,8.3]) |
| **Use e-cigarette in previous 6 months** | | | |
| Did use e-cigarette | 671/4206  (16.0% [14.9,17.1]) | 126/835  (15.1% [12.8,17.7]) | 545/3371  (16.2% [15.0,17.4]) |

# Appendix 7: Sample characteristics among participants present at 36-month post-baseline follow-up in the Health4Life trial, grouped by relative Low SES versus Mid to High SES levels

|  | Low SES (N = 571) | Mid to High SES (N = 3518) |
| --- | --- | --- |
| **Age** (mean [SD]) | 15.70 (0.55) | 15.71 (0.65) |
| **Gender identity** (N [%]) | | |
| Male | 326 (57.2%) | 1641 (46.8%) |
| Female | 215 (37.7%) | 1708 (48.7%) |
| Non-binary | 17 (3.0%) | 101 (2.9%) |
| Prefer not to say | 12 (2.1%) | 59 (1.7%) |
| **Psychological distress** (N [%]) | | |
| No | 441 (81.7%) | 2662 (79.8%) |
| Yes | 99 (18.3%) | 674 (20.2%) |
| **State** (N [%]) |  |  |
| NSW | 358 (62.7%) | 1874 (53.3%) |
| QLD | 127 (22.2%) | 1015 (28.9%) |
| WA | 86 (15.1%) | 629 (17.9%) |
| **Socio-economic status** (N [%]) | | |
| Low | 571 (100%) | 0 (0%) |
| Mid | 0 (0%) | 1529 (43.5%) |
| High | 0 (0%) | 1989 (56.5%) |
| **Geographical location** (N [%]) | | |
| Major city | 497 (87.0%) | 3235 (92.0%) |
| Regional | 74 (13.0%) | 283 (8.0%) |

# Appendix 8: Risk behaviours among participants present at 36-month post-baseline follow-up in the Health4Life trial, grouped by relative Low SES versus Mid to High SES levels (unadjusted values)

|  | Low SES (N = 571) | Mid to High SES (N = 3518) |
| --- | --- | --- |
| **SSB consumption** | | |
| More than 5 cups (N [% [95% CI]]) | 76/550  (13.8% [11.2,17.0]) | 322/3386  (9.5% [8.6,10.5]) |
| **Fruit intake** | | |
| Insufficient fruit (N [% [95% CI]]) | 210/551  (38.1% [34.2,42.2]) | 1169/3386  (34.5% [32.9,36.1]) |
| **Vegetable intake** | | |
| Insufficient vegetable (N [% [95% CI]]) | 468/551  (84.9% [81.7,87.7]) | 2952/3386  (87.2% [86.0,88.3]) |
| **Discretionary food intake** | | |
| Excessive (N [% [95% CI]]) | 187/513  (36.5% [32.4,40.7]) | 1317/3173  (41.5% [39.8,43.2]) |
| **Full standard drink in previous 6 months** | | |
| Did consume full standard drink | 135/547  (24.7% [21.2,28.5]) | 1024/3355  (30.5% [30.0,32.1]) |
| **Binge drink in previous 6 months** | | |
| Did binge drink | 54/546  (9.9% [7.7,12.7]) | 456/3340  (13.7% [12.5,14.9]) |
| **Tobacco smoking in previous 6 months** | | |
| Did smoke tobacco cigarette | 50/539  (9.3% [7.1,12.0]) | 250/3347  (7.5% [6.6,8.4]) |
| **Use e-cigarette in previous 6 months** | | |
| Did use e-cigarette | 73/540  (13.5% [10.9,16.7]) | 528/3343  (15.8% [14.6,17.1]) |

# Appendix 9: Sample characteristics of participants present at 36-month post-baseline follow-up in the Health4Life trial, grouped by geographical location

|  | Regional (N = 399) | Major City (N = 4046) |
| --- | --- | --- |
| **Age** (mean [SD]) | 15.73 (0.5) | 15.71 (0.65) |
| **Gender identity** (N [%]) |  |  |
| Male | 229 (57.5%) | 1943 (48.2%) |
| Female | 152 (38.2%) | 1913 (47.4%) |
| Non-binary | 10 (2.5%) | 113 (2.8%) |
| Prefer not to say | 7 (1.8%) | 66 (1.6%) |
| **Psychological distress** (N [%]) |  |  |
| No | 286 (79.7%) | 3063 (79.9%) |
| Yes | 73 (20.3%) | 771 (20.1%) |
| **State** (N [%]) |  |  |
| NSW | 212 (53.1%) | 2186 (54.0%) |
| QLD | 187 (46.9%) | 1212 (30.0%) |
| WA | 0 (0%) | 648 (16.0%) |
| **Socio-economic status** (N [%]) |  |  |
| Low | 74 (20.7%) | 497 (13.3%) |
| Mid | 173 (48.5%) | 1356 (36.3%) |
| High | 110 (30.8%) | 1879 (50.4%) |
| **Geographical location** (N [%]) |  |  |
| Major city | 0 (0%) | 4046 (100%) |
| Regional | 399 (100%) | 0 (0%) |

# Appendix 10: Risk behaviours among participants present at 36-month post-baseline follow-up in the Health4Life trial, grouped by geographical location (unadjusted values)

|  | Regional (N = 399) | Major City (N = 4046) |
| --- | --- | --- |
| **SSB consumption** | | |
| Excessive/more than 5 cups (N [% [95% CI]]) | 47/373  (12.6% [9.6,16.4]) | 397/3899  (10.2% [9.3,11.2]) |
| **Fruit intake** | | |
| Insufficient fruit (N [% [95% CI]]) | 125/373  (33.5% [28.9,38.4]) | 1383/3900  (35.5% [34.0,37.0]) |
| **Vegetable intake** | | |
| Insufficient vegetable (N [% [95% CI]]) | 309/373  (82.8% [78.7,86.3]) | 3399/3900  (87.2% [86.1,88.2]) |
| **Discretionary food intake** | | |
| Excessive (N [% [95% CI]]) | 117/327  (35.8% [30.8,41.1]) | 1504/3633  (41.4% [39.8,43.0]) |
| **Full standard drink in previous 6 months** | | |
| Did consume full standard drink | 141/366  (38.5% [33.7,43.6]) | 1122/3859  (29.1% [27.7,30.5]) |
| **Binge drink in previous 6 months** | | |
| Did binge drink | 74/365  (20.3% [16.5,24.7]) | 495/3842  (12.9% [11.9,14.0]) |
| **Tobacco smoking in previous 6 months** | | |
| Did smoke tobacco cigarette | 50/363  (13.8% [10.6,17.7]) | 284/3849  (7.4% [6.6,8.2]) |
| **Use e-cigarette in previous 6 months** | | |
| Did use e-cigarette | 69/363  (19.0% [15.3,23.3]) | 602/3843  (15.7% [14.6,16.8]) |

# Appendix 11: Prevalence ratios with 95% confidence intervals (CI) for each risk behaviour among 4445 participants present at 36-month post-baseline follow-up in the Health4Life trial

|  | Prevalence Ratio | Lower 95% CI | Upper 95% CI |
| --- | --- | --- | --- |
| **SSB consumption (more than 5 cups)** | | | |
| *More advantaged (ref) v Disadvantaged | 1.17 | 0.90 | 1.52 |
| Major city (ref) v Regional area | 1.24 | 0.75 | 2.05 |
| Mid to High SES (ref) v Low SES | 1.24 | 0.94 | 1.64 |
| **Insufficient fruit intake (<2 servings of fruit/day)** | | | |
| *More advantaged (ref) v Disadvantaged | 1.03 | 0.91 | 1.16 |
| Major city (ref) v Regional area | 0.88 | 0.70 | 1.11 |
| Mid to High SES (ref) v Low SES | 1.09 | 0.96 | 1.25 |
| **Insufficient vegetable intake (<5 servings of vegetables/day)** | | | |
| *More advantaged (ref) v Disadvantaged | 0.98 | 0.96 | 1.01 |
| Major city (ref) v Regional area | 0.96 | 0.92 | 1.01 |
| Mid to High SES (ref) v Low SES | 0.99 | 0.97 | 1.02 |
| **Excessive discretionary food consumption (≥1/day)** | | | |
| *More advantaged (ref) v Disadvantaged | 0.84 | 0.76 | 0.94 |
| Major city (ref) v Regional area | 0.88 | 0.74 | 1.04 |
| Mid to High SES (ref) v Low SES | 0.87 | 0.77 | 0.99 |
| **Consumed full standard alcoholic drink (in previous 6-months)** | | | |
| *More advantaged (ref) v Disadvantaged | 0.84 | 0.71 | 1.00 |
| Major city (ref) v Regional area | 1.41 | 1.00 | 1.97 |
| Mid to High SES (ref) v Low SES | 0.78 | 0.65 | 0.93 |
| **Binge drank alcohol (in previous 6-months)** | | | |
| *More advantaged (ref) v Disadvantaged | 0.76 | 0.58 | 1.01 |
| Major city (ref) v Regional area | 1.77 | 1.07 | 2.93 |
| Mid to High SES (ref) v Low SES | 0.68 | 0.50 | 0.92 |
| **Tobacco use (any use in previous 6-months)** | | | |
| *More advantaged (ref) v Disadvantaged | 1.32 | 0.97 | 1.79 |
| Major city (ref) v Regional area | 2.06 | 1.18 | 3.60 |
| Mid to High SES (ref) v Low SES | 1.23 | 0.89 | 1.71 |
| **E-cigarette use (any use in previous 6-months)** | | | |
| *More advantaged (ref) v Disadvantaged | 0.89 | 0.71 | 1.12 |
| Major city (ref) v Regional area | 1.24 | 0.83 | 1.87 |
| Mid to High SES (ref) v Low SES | 0.85 | 0.66 | 1.09 |

**More advantaged versus disadvantaged were included in a separate model to comparing geographical location (major city v regional area) and socioeconomic status (mid to high v low SES) due to the overlap of the groupings.*

Note: Prevalence ratios with 95% CIs that did not cross 1 were considered significant.

# Forest plots for each primary outcome

*
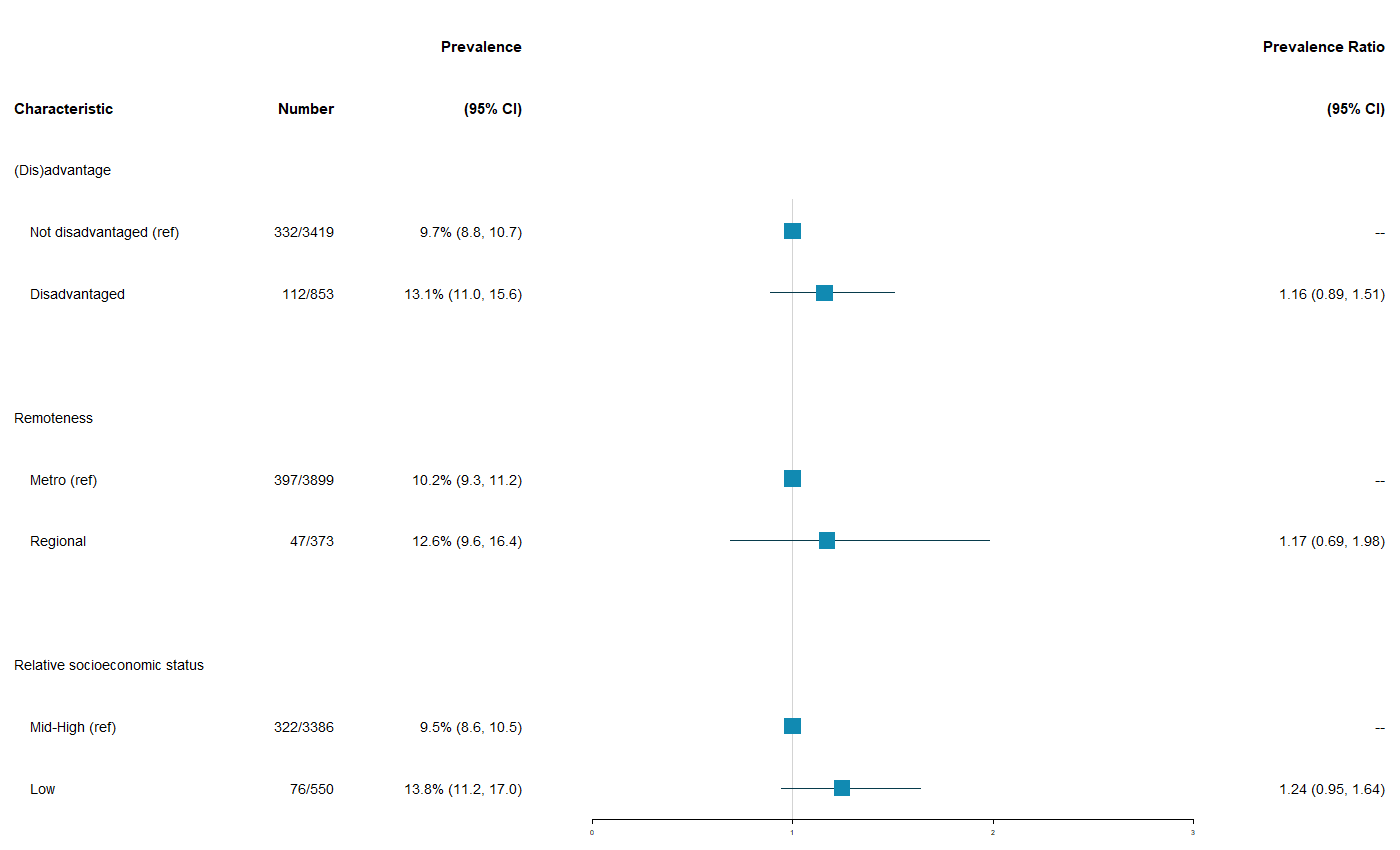
*

## Appendix 12: Excessive SSB consumption forest plot

*
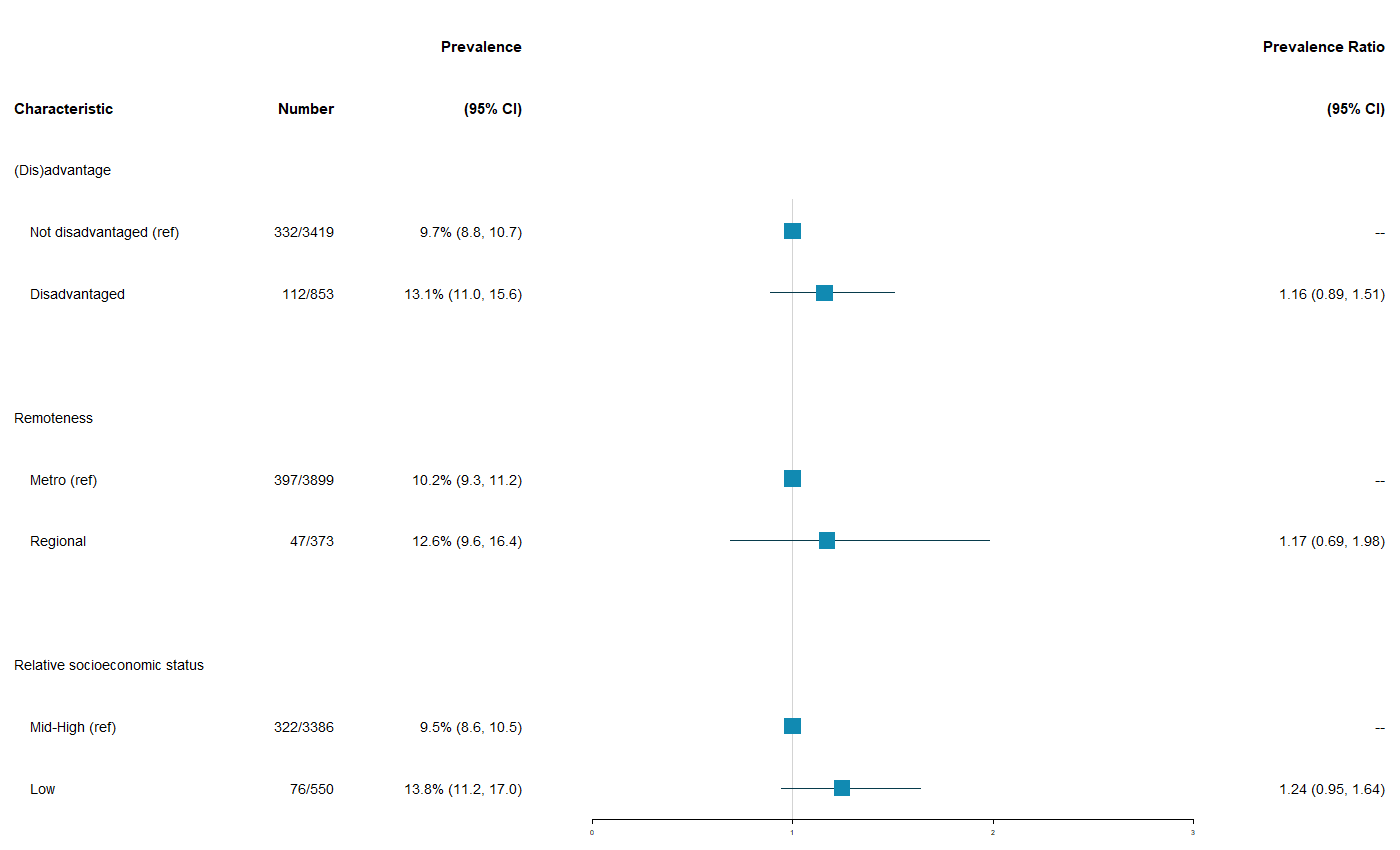
*

*
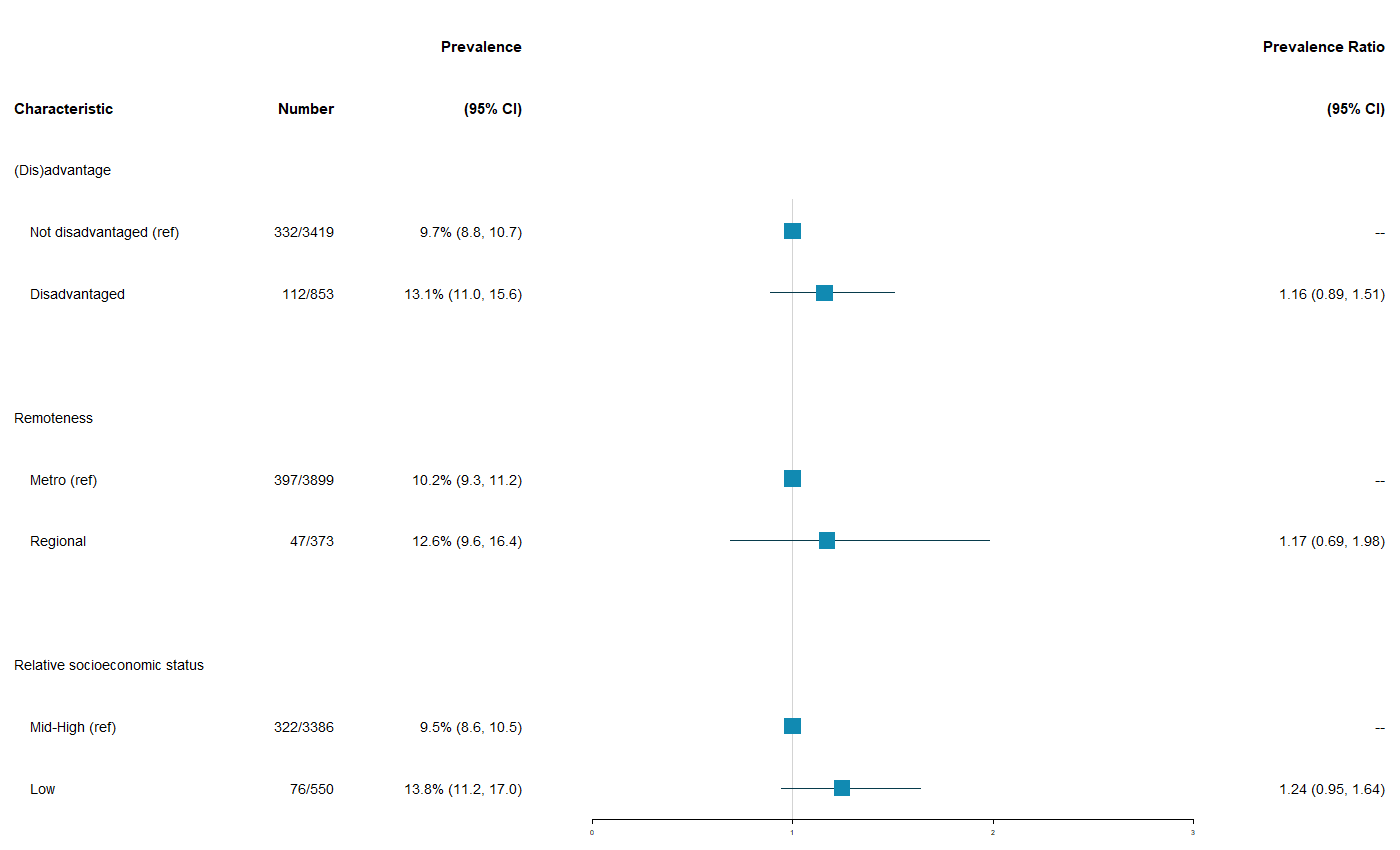
*


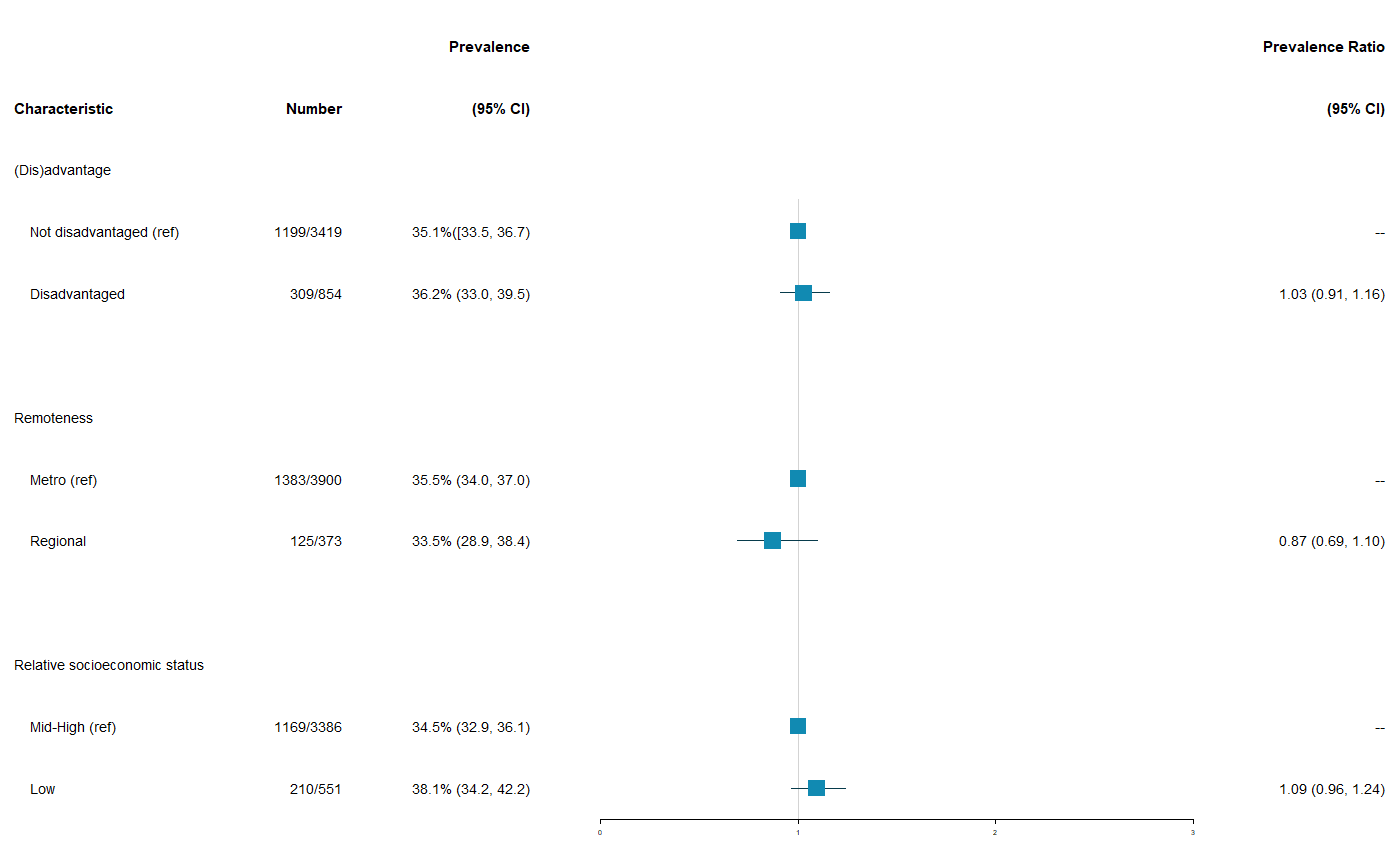


## Appendix 13: Insufficient fruit intake forest plot


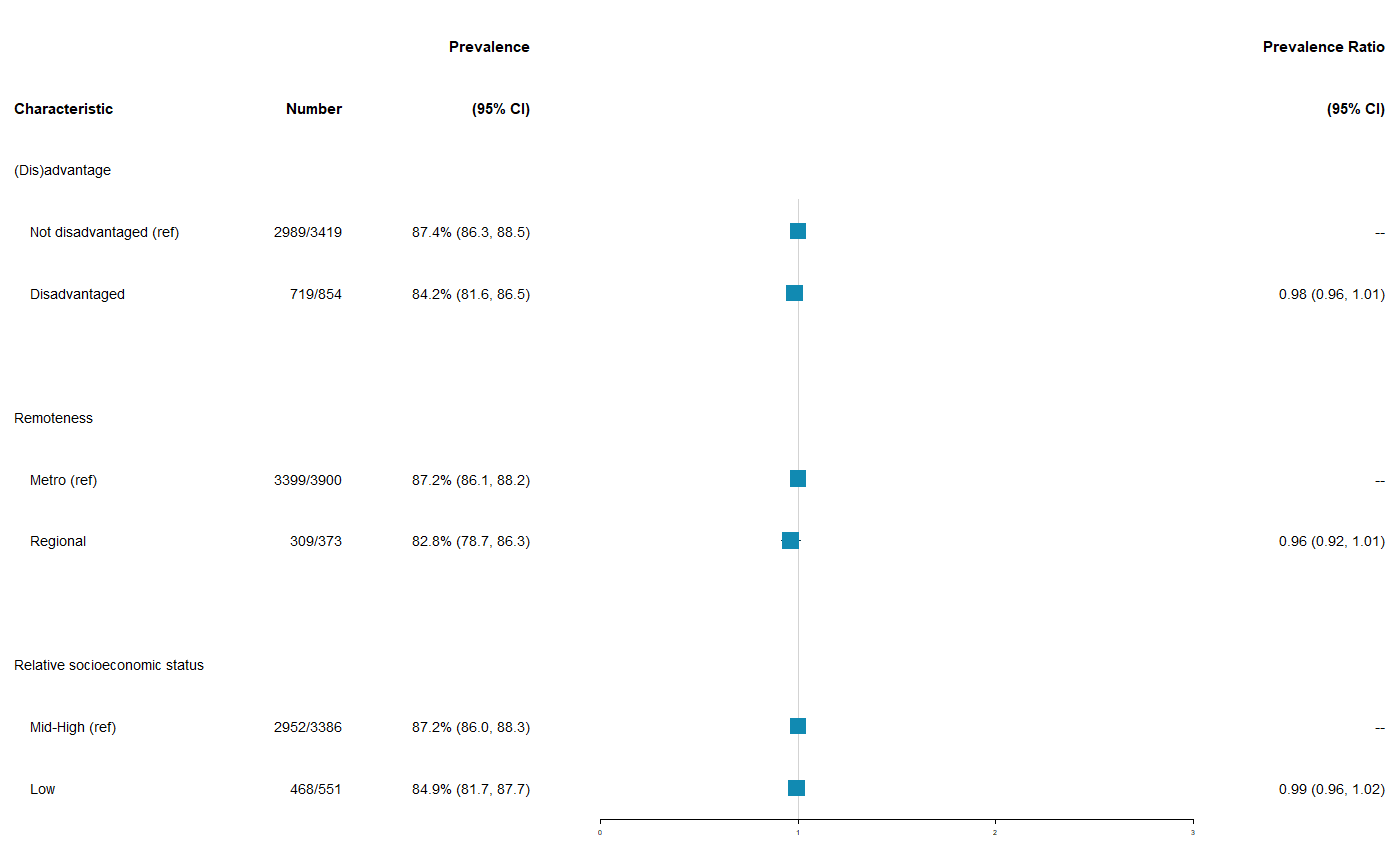


## Appendix 14: Insufficient vegetable intake forest plot


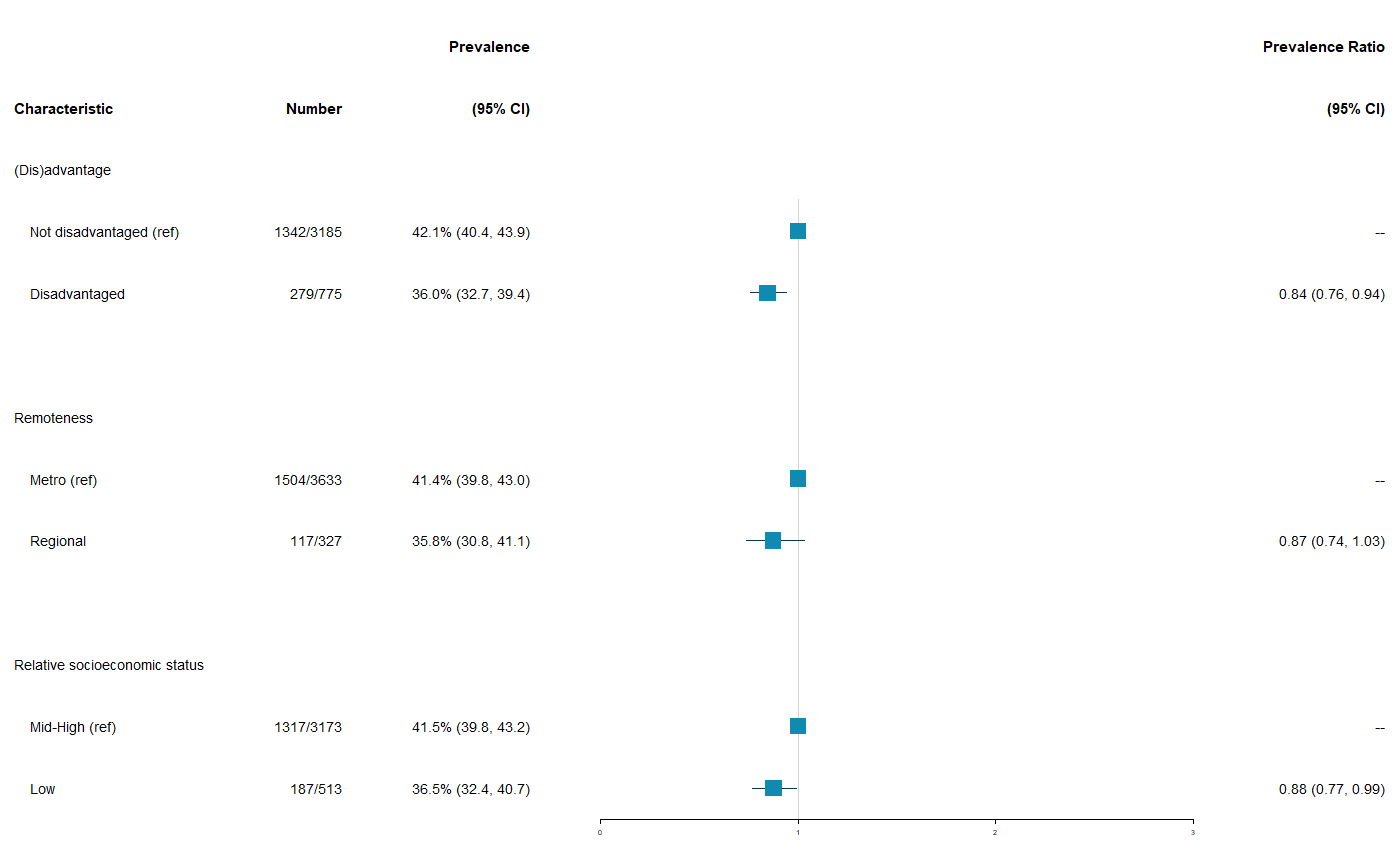


## Appendix 15: Excessive discretionary food consumption forest plot


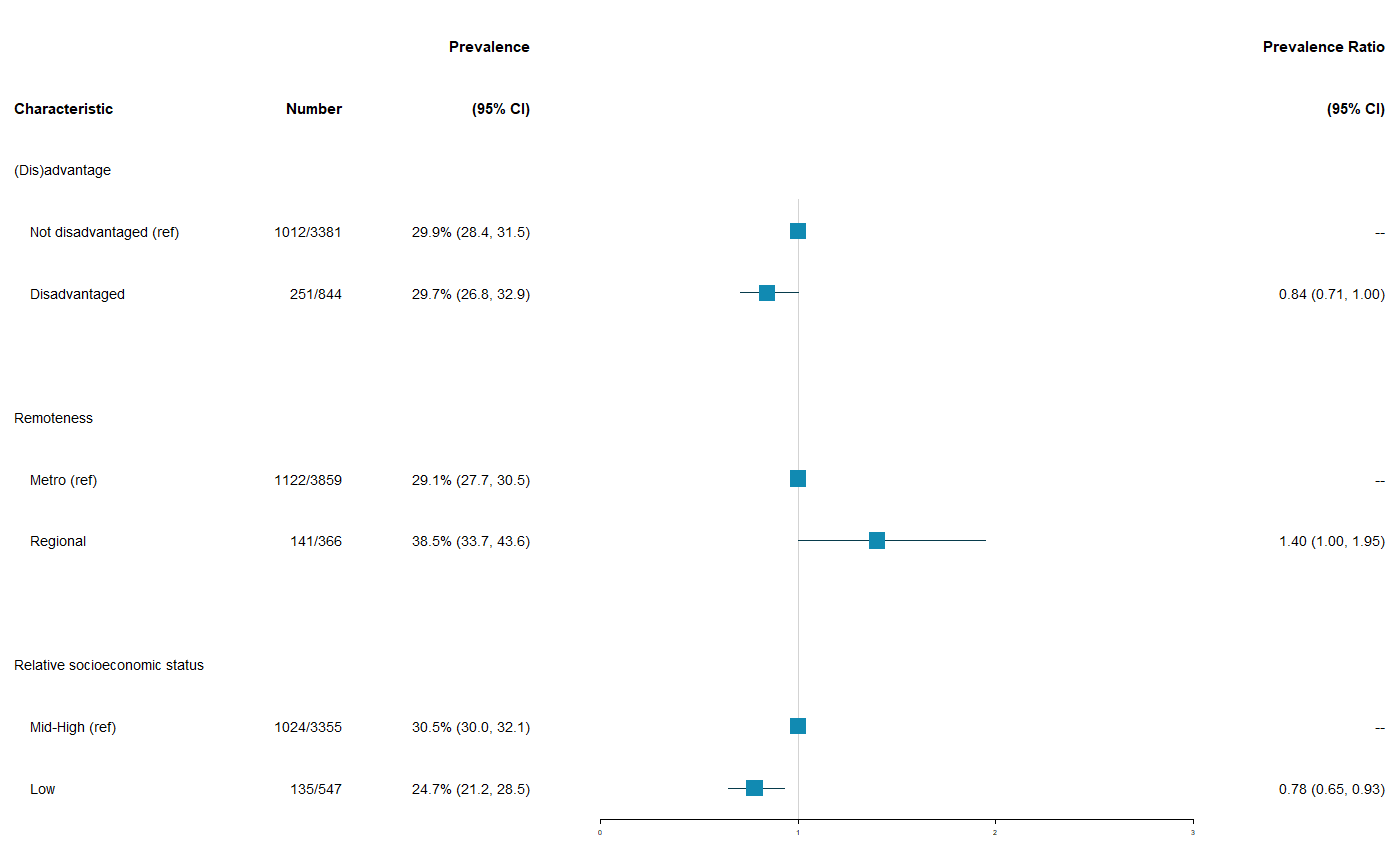


## Appendix 16: Consumed full standard drink of alcohol in previous 6-months forest plot


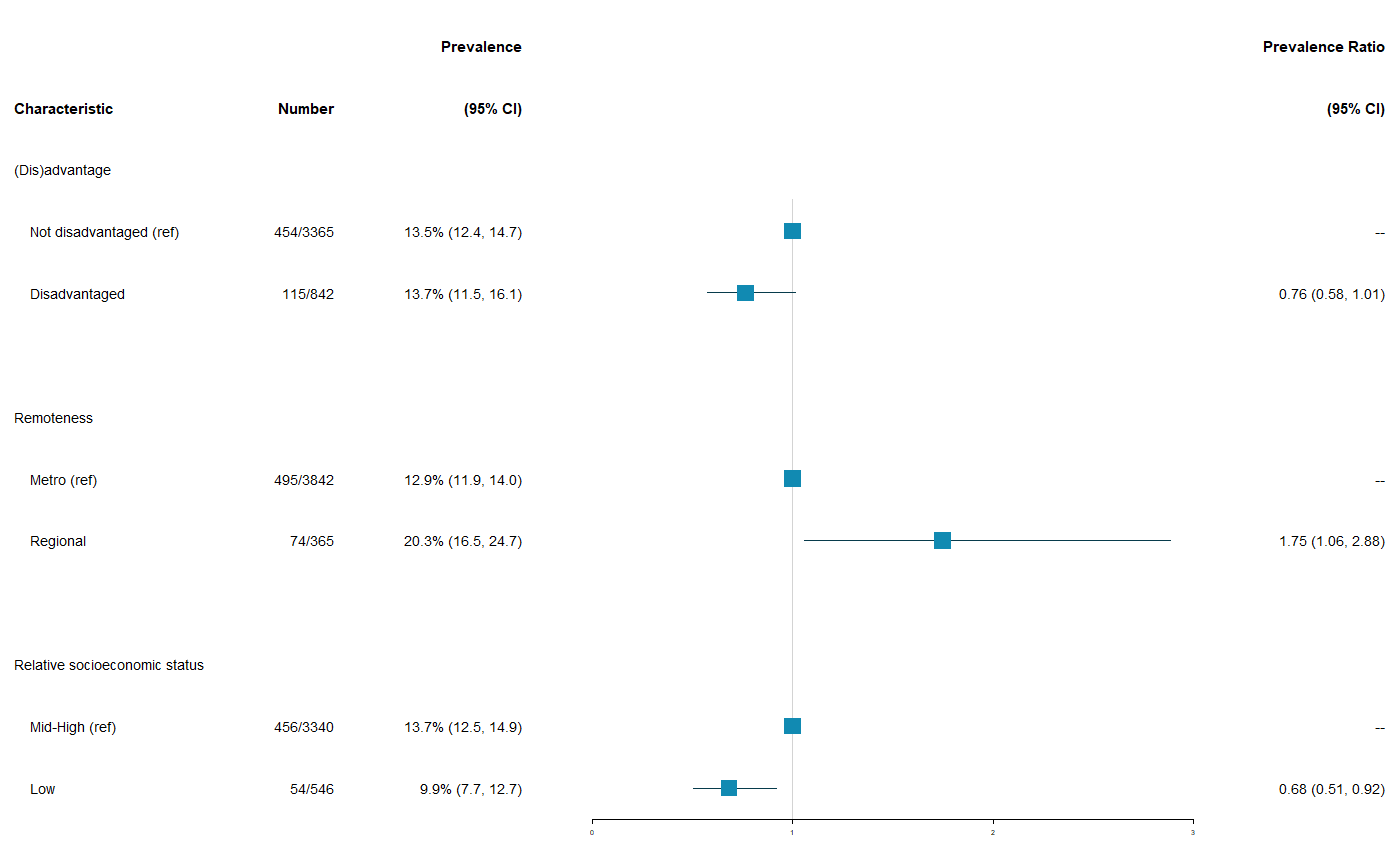

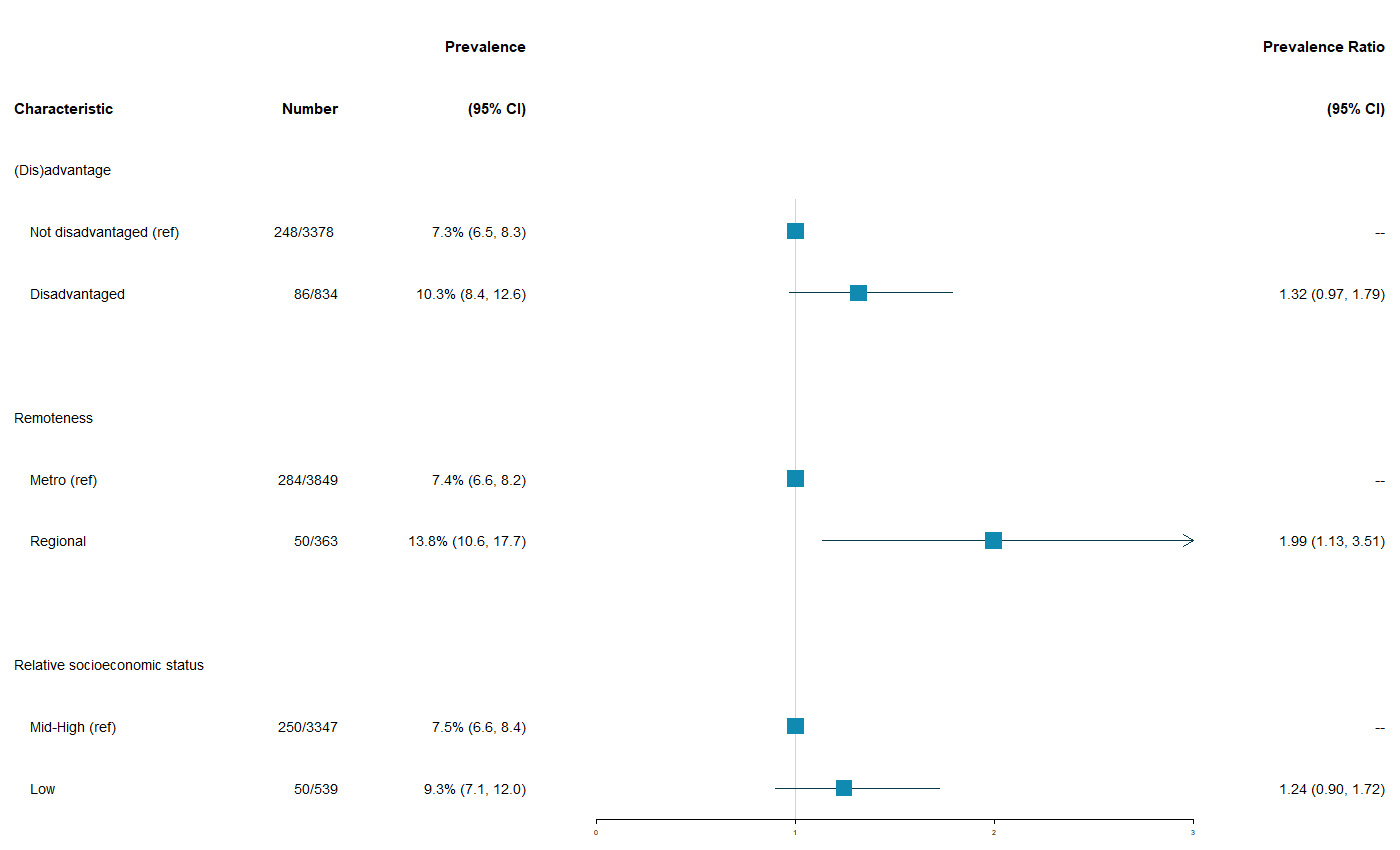


## Appendix 14: Binge drank alcohol in previous 6-months forest plot

## Appendix 17: Tobacco smoking in previous 6-months forest plot


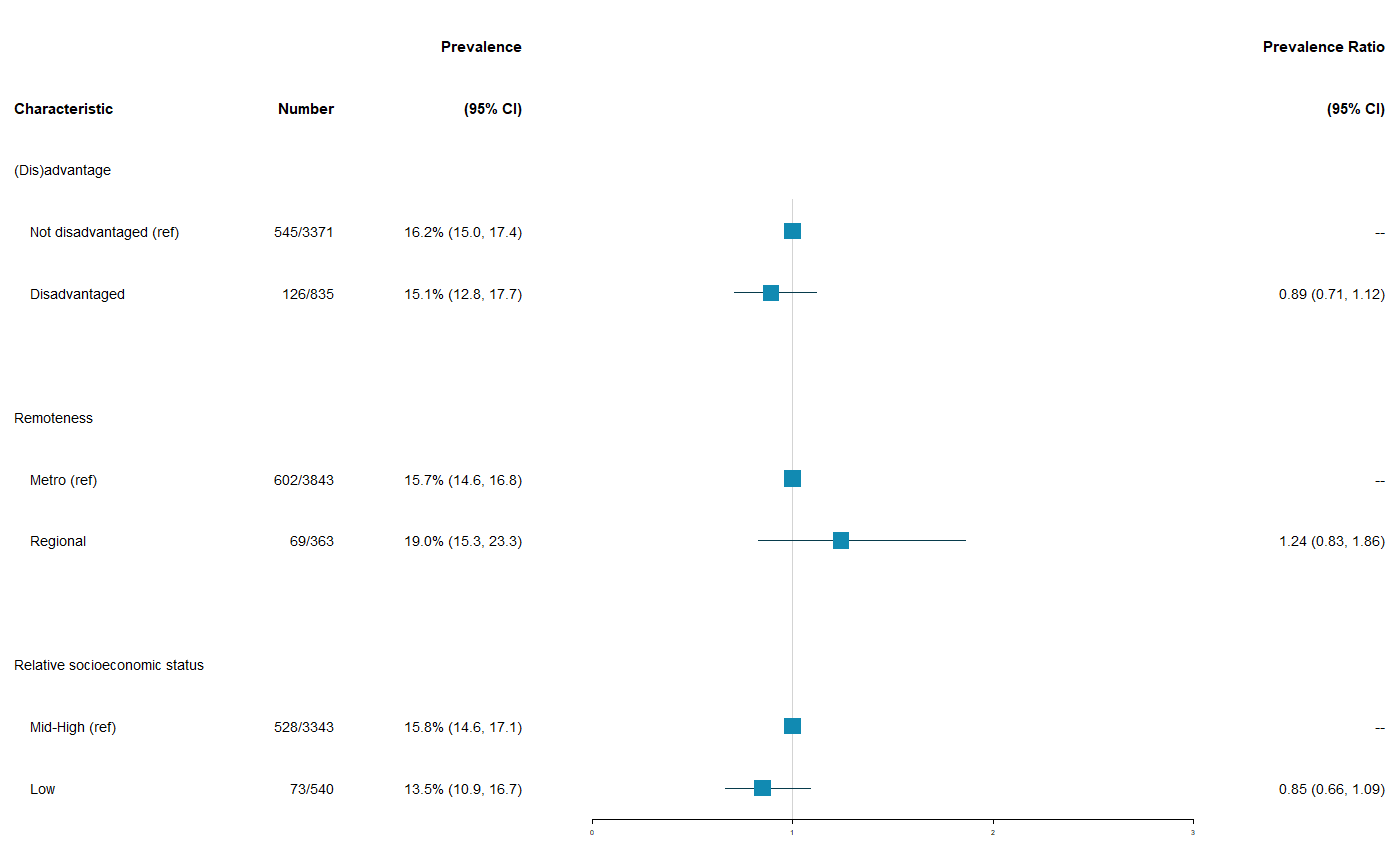


## Appendix 18: E-cigarette use in previous 6-months forest plot

**References**

1. Champion KE, Chapman C, Gardner LA, Sunderland M, Newton NC, Smout S, Thornton LK, Hides L, McBride N, Allsop SJ, Mills K. Lifestyle risks for chronic disease among Australian adolescents: a cross-sectional survey. Med J Aust. 2022;216(3):156-7.

1. Due to higher missing responses on the discretionary food intake question at 36-months among adolescents (11%), simple logistic regressions investigating differences in missingness on this variable were conducted. The results reported no significant differences in baseline discretionary food scores between those with and without missing data at 36 months (OR = 0.98, 95% CI: 0.88–1.09, p = 0.7). However, lower SES participants were less likely to have missing data at 36-months compared to mid-to-high SES participants (OR = 0.79, 95% CI: 0.68–0.91, p = 0.001). Additionally, regional adolescents were less likely to have missing data on discretionary food at 36-months than those in metropolitan areas (OR = 0.58, 95% CI: 0.68–0.91, p < 0.001). [↑](#footnote-ref-1)
